# DDX3 Regulates the Cap‐Independent Translation of the Japanese Encephalitis Virus via Its Interactions with PABP1 and the Untranslated Regions of the Viral Genome

**DOI:** 10.1002/advs.202502493

**Published:** 2025-05-08

**Authors:** Chenxi Li, Linjie Zhang, Chenyang Tang, Xuan Chen, Jing Shi, Qingyu Li, Xue Jiao, Jinyao Guo, Bin Wang, Kefan Bu, Abdul Wahaab, Yuguo Yuan, Ming‐an Sun, Yanhua Li

**Affiliations:** ^1^ College of Veterinary Medicine Yangzhou University Yangzhou Jiangsu 225009 China; ^2^ Jiangsu Co‐innovation Center for Prevention and Control of Important Animal Infectious Diseases and Zoonoses Yangzhou University Yangzhou Jiangsu 225009 China; ^3^ The Center for infectious Disease Dynamics and the Huck Institutes of the Life Sciences Pennsylvania State University, University Park State College PA 16802 USA; ^4^ Department of Microbiology and Immunology University of Texas Medical Branch Galveston TX 77 555 USA

**Keywords:** cap‐independent translation, DDX3, Japanese encephalitis virus

## Abstract

The translation of global cellular proteins is almost completely repressed in cells with flavivirus infection, while viral translation remains efficient. The mechanisms of flaviviruses evade host translational shutoff are largely unknown. Here, it is found that Japanese encephalitis virus (JEV) can adopt cap‐independent (CI) translation to escape the host translational shutoff. Furthermore, the elements DB2 and sHP‐SL within 3′UTR are involved in the regulation of CI translation, which is conserved in the genus *Orthoflavivirus*. By RNA affinity purification and mass spectrometry analysis, cellular DEAD‐box protein 3 (DDX3) and poly(A)‐binding protein 1 (PABP1) are identified as key factors in regulating CI translation of JEV via their interactions with DB2 and sHP‐SL RNA structures. Mechanistically, it is revealed that DDX3 binds to both 5′UTR and 3′UTR of the JEV genome to establish a closed‐loop architecture and recruit eIF4G/eIF4A to form the DDX3/PABP1/eIF4G/eIF4A tetrameric complex via its interaction with PABP1, thereby recruiting the ribosomal 43S preinitiation complex (PIC) to the 5′‐end of the JEV genome to start translation. These findings demonstrate a noncanonical translation strategy employed by JEV and further reveal the regulatory roles of DDX3 and PABP1 in this mechanism. These results expand the knowledge of the translation initiation regulation in flaviviruses under the state of host translational shutoff, which provides a conserved antiviral target against *orthoflavivirus*.

## Graphical abstract

Japanese encephalitis virus (JEV) employs a cap‐independent (CI) translation strategy to evade host translational shutoff. During the shut‐off of host cellular canonical translation, DEAD‐box protein 3 (DDX3) bind to JEV UTRs to establish a closed‐loop architecture, and its interaction with poly(A)‐binding protein 1 (PABP1) to form DDX3/PABP1/eIF4G/eIF4A tetrameric complex, thereby recruiting the ribosomal 43S preinitiation complex (PIC) to initiate translation.

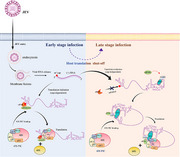

## 1 Introduction

Japanese encephalitis virus (JEV) is a member of the genus *Orthoflavivirus* in the *Flaviviridae* family which includes many important zoonotic pathogens, such as Zika virus (ZIKV), Dengue virus (DENV), West Nile virus (WNV), and Tick‐borne encephalitis virus (TBEV).^[^ ^1^, ^2^ ^]^ As an arthropod‐borne virus, JEV is primarily transmitted through mosquito vectors from vertebrate‐amplifying hosts to susceptible hosts,^[^ ^3^, ^4^ ^]^ and it causes encephalitis in humans, horses, and piglets and abortion and orchitis in breeding pigs.^[^ ^5^, ^6^ ^]^ Pigs and birds serve as amplifying or reservoir hosts, which play essential roles in the bird‐associated wild transmission cycle and the pig‐associated rural domestic transmission cycle, respectively.^[^ ^7^ ^]^ Humans and horses are considered the dead‐end hosts for JEV. The JEV genome comprises a single open reading frame (ORF) flanked by 5′ and 3′ untranslated regions (UTRs). The 5′ UTR with a type I cap structure is ≈100 nucleotides, while the 3′ UTR lacking a poly(A) tail is ≈570 nucleotides. High‐order RNA structures formed in 5′UTR and 3′UTR function as *cis*‐acting elements required for viral RNA synthesis and viral protein translation.^[^ ^8^ ^]^ JEV genome could directly serve as mRNA to translate a single polyprotein which is post‐translationally processed by viral and host proteinases to generate three structural proteins (envelope [E], pre‐membrane [prM], and capsid [C]) and seven nonstructural proteins (NS1, NS2A, NS2B, NS3, NS4A, NS4B, and NS5).^[^ ^9^, ^10^ ^]^ Based on the coding sequence of the E protein, JEV is phylogenetically classified into five genotypes (genotype I to V), and the prevalent strains mainly belong to genotype I (GI) and genotype III (GIII).^[^ ^11^, ^12^ ^]^

The translation initiation is the most important phase for the regulation of gene expression. In eukaryotes, the initiation of protein synthesis for most mRNAs generally occurs via the canonical cap‐dependent mechanism.^[^ ^13^ ^]^ To initiate cap‐dependent (CD) translation, the eukaryotic initiation factor 4E (eIF4E) first recognizes and binds to the m^7^G(5′)ppp(5′)N cap structure at the 5′ end of mRNA, and then recruits an adaptor protein (eIF4G) and a RNA helicase complex (eIF4A and co‐factor eIF4B) to form eIF4F cap‐binding complex (eIF4E, eIF4G, and eIF4A), which further recruits the ribosomal 43S preinitiation complex (PIC) composed of a small 40S ribosomal subunit, the translation initiation factors (eIF1, eIF1A, eIF3, eIF5) and the ternary eIF2‐GTP‐Met‐tRNAi complex, onto the mRNA.^[^ ^14^ ^]^ Subsequently, the 43S PIC scans mRNAs in the 5′ to 3′ direction for the AUG start codon and recruits a 60S large ribosomal subunit to form an 80S monosome to initiate polypeptide synthesis.^[^ ^15^ ^]^ Besides the necessary eIFs and ribosome subunits, various host proteins also participate in translation initiation. As a member of the DEAD (Asp‐Glu‐Ala‐Asp) box family of RNA helicases, the multifunctional DEAD‐box protein 3 (DDX3) contributes to the translation initiation process. DDX3 may accomplish modulation of cellular mRNA translation by its interactions with mRNA, poly(A)‐binding protein 1 (PABP1), and initiation factors such as eIF2, eIF4E, and eIF4G.^[^ ^16^ ^]^ In addition, DDX3 also interacts with the components of the 43S PIC, such as eIF3 and the 40S ribosomal subunit, thereby mediating the recruitment of 43S PIC to mRNAs.^[^ ^17^, ^18^ ^]^ Even though DDX3 was reported as a general translation initiation factor, DDX3 appears to only promote the translation of a subset of selected mRNAs with specific RNA structures at their 5′‐end. Recently, DDX3 was demonstrated to enhance viral translation of JEV,^[^ ^19^ ^]^ Hepatitis C virus (HCV),^[^ ^16^ ^]^ and Foot‐and‐mouth disease virus^[^ ^20^ ^]^ for efficient propagation. However, the mechanistic basis has not been fully understood.

Since viruses cannot replicate outside of living cells, the expression of viral proteins exclusively relies on host translation apparatus. However, as an antiviral strategy, the CD translation in virus‐infected eukaryotic cells is generally shut down to prevent the synthesis of viral proteins.^[^ ^21^, ^22^ ^]^ To overcome host shutoff or to compete for translational resources, viruses have evolved diverse CI translation mechanisms to support the robust expression of viral proteins. For instance, the VPg proteins of murine norovirus and feline calicivirus which serve as a substitute for the 5′cap structure allow viral mRNA binding to eIF4E or even directly to eIF4G.^[^ ^23^, ^24^ ^]^ The internal ribosomal entry sites (IRESs) harbored by HCV,^[^ ^25^ ^]^ bovine viral diarrhea virus (BVDV),^[^ ^26^ ^]^ classical swine fever virus^[^ ^27^ ^]^ and human immunodeficiency virus,^[^ ^28^ ^]^ could directly recruit ribosomes to the internal region of viral mRNAs, omitting the need for eIF4E and eIF4G. Interestingly, several positive‐strand RNA plant viruses, such as barley yellow dwarf virus,^[^ ^29^ ^]^ panicum mosaic virus,^[^ ^30^ ^]^ and tomato bushy stunt virus,^[^ ^31^ ^]^ could employ a CI translation element (CITE) residing in the 3′UTR to initiate translation. This mechanism of viral translation initiates at the 5′‐end with the assistance of viral 3′UTR. Notably, the CD and CI translations are not mutually exclusive and may synergically enhance viral translation.

In response to cellular stress triggered by flavivirus infection, cellular protein translation is often controlled at the rate‐limiting step of initiation.^[^ ^32^, ^33^, ^34^ ^]^ The repression of the initiation step of CD translation was documented for DENV, WNV, TBEV, duck Tembusu virus (DTMUV), and ZIKV.^[^ ^33^, ^34^ ^]^ Despite host translational shutoff, the synthesis of flavivirus proteins remains efficient in supporting viral replication. DENV employs a novel non‐IRES‐mediated non‐canonical translation mechanism that requires the interaction between DENV 5′UTR and 3′UTR,^[^ ^35^ ^]^ while the 5′UTR of ZIKV without a type‐I cap structure harbored IRES activity which directly initiates CI translation.^[^ ^36^ ^]^ In addition, *cis*‐acting elements in DTMUV 3′UTR were shown to play crucial regulatory roles in CI translation.^[^ ^33^ ^]^ Thus, flaviviruses might have various strategies to regulate non‐canonical translation initiation. Of note, experimental evidence supporting the non‐canonical translation of JEV is lacking. In this study, we found that JEV can be rescued using genomic RNA without a cap structure via CI translation in cells of different species. JEV 5′UTR lacks IRES or IRES‐like activity, while 5′UTR could initiate the CI translation in the presence of 3′UTR. Mechanistically, we revealed that DB2 and sHP‐SL elements within 3′UTR play decisive roles in the regulation of CI translation via their interactions with DDX3 and PABP1. As a translation initiation factor, DDX3 anchors to both 5′UTR and 3′UTR of the JEV genome, forming the DDX3/PABP1/eIF4G/eIF4A tetrameric complex via its interaction with PABP1, thereby recruiting the 43S PIC to the 5′‐end of the JEV genome and allowing translation initiation. These results provide new insights for the understanding of the translational initiation regulation of flaviviruses.

## 2 Results

### 2.1 JEV Adopts a CI Translation Strategy to Evade Host Shutoff

**Figure 1:**
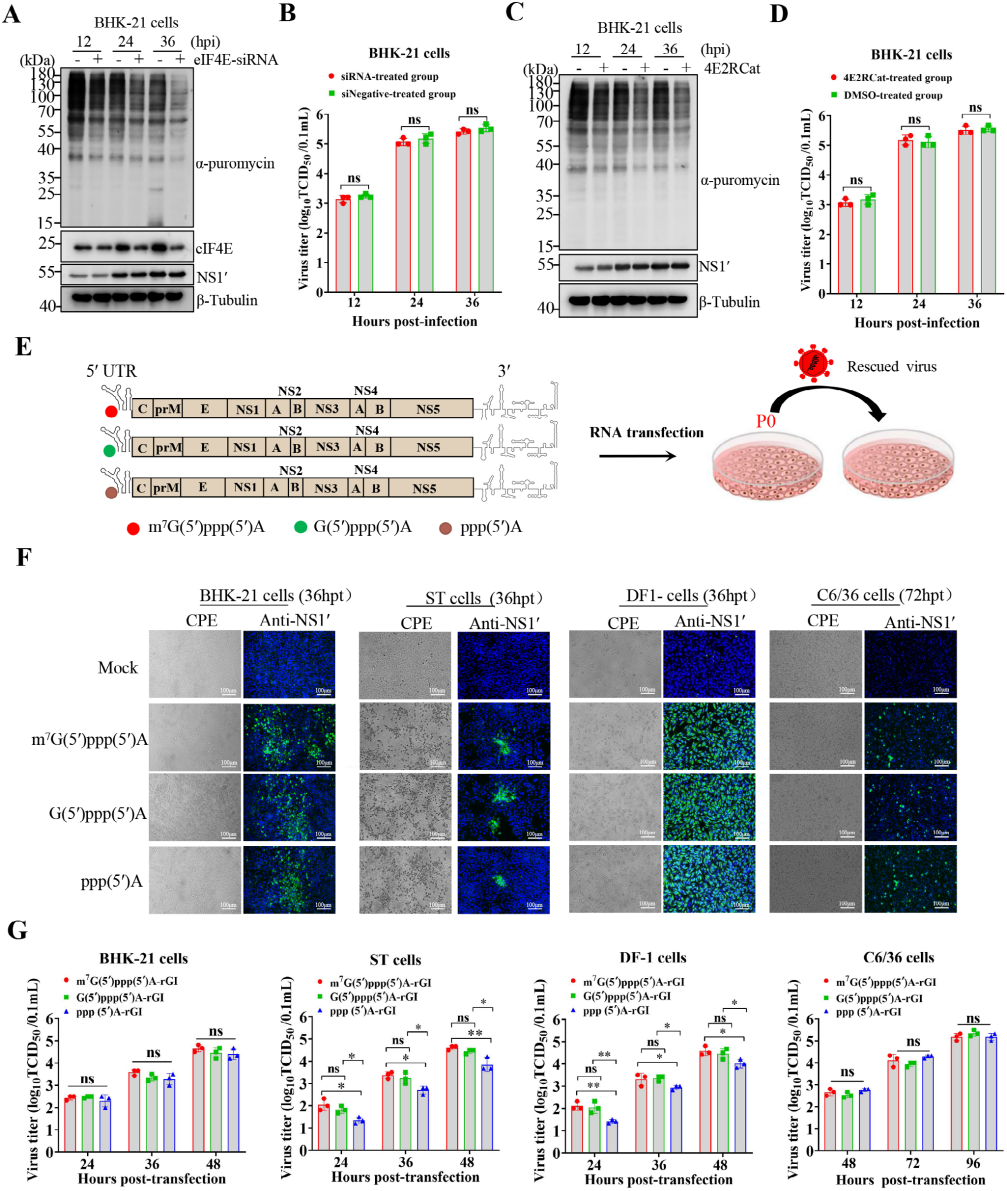
Both CD and CI translation initiation strategies are involved in the expression of JEV proteins. A–D) BHK‐21 cells were respectively transfected with 100 pmol of a mixture of eIF4E‐specific siRNAs (Table S3, Supporting Information) or treated with 20 µm 4E2RCat for 12 h, and then infected with JEV at an MOI of 0.1. At different time points of post‐infection, the cells were labeled with puromycin for 30 min and harvested to analyze puromycin incorporation (A,C). The viral titers in culture supernatants of BHK‐21 cells treated with eIF4E‐specific siRNAs (B) or 4E2RCat (D) were measured by TCID_50_ assay (*n* = 3). E) Schematic diagram of the virus rescue using JEV RNA transcripts modified with three different 5′termini: m^7^G(5′)ppp(5′)A, G(5′)ppp(5′)A and ppp(5′)A. F) Cytopathic effects and immunofluorescence assay of cells transfected with full‐length viral RNA transcripts with different 5′ termini. G) The viral titers in culture supernatants of BHK‐21, ST, and DF‐1 cells transfected with 2 µg RNA transcripts at 24, 36, and 48 hpt, and the viral titers in culture supernatants of C6/36 cells transfected with 2 µg RNA transcripts at 48, 72, and 96 hpt, were measured by TCID_50_ assay on BHK‐21 cells (*n* = 3). *, *p* < 0.05; **, *p* < 0.01; ns, no statistical difference. Data are presented as mean ± standard deviation (SD) of three independent experiments and tested by Student's *t*‐test (B, D, and G).

Since their genomes harbor a type I cap structure at the 5′end, flaviviruses are supposed to initiate viral translation via the cap‐dependent mechanism.^[^ ^37^ ^]^ However, the synthesis of viral polyproteins remains efficient when the cap‐dependent host cellular translation is suppressed at the level of translation initiation during flavivirus infection.^[^ ^34^, ^37^ ^]^ Here, puromycin incorporation assays confirmed that JEV infection could induce host translational shutoff in various susceptible vertebrate/mosquito cells, including mosquito C6/36, chicken DF‐1, porcine ST, and baby hamster BHK‐21 cells (Figure S1A–D, Supporting Information), which used as in vitro models of mosquito, bird, pig and rodent hosts, respectively. In contrast, the expression level of NS1′ gradually increased as infection went on, suggesting that JEV translation could evade the shutoff of protein synthesis in distinct hosts. In BHK‐21 and ST cells, downregulation of CD translation by reduction of eIF4E expression or interfering with the interaction between eIF4E and eIF4G using the inhibitor 4E2RCat had no obvious effect on NS1′ expression and viral yields (**Figure** **1**A–D; Figure S1E–H, Supporting Information), suggesting that JEV translation could be initiated with other mechanisms. To further confirm whether type I cap structure is indispensable for the translation initiation and virus recovery of JEV, three types of genomic RNA of the GI JEV with different 5′ends illustrated in Figure 1E were synthesized for virus recovery. The JEV RNA transcripts were respectively transfected into four cell lines derived from different host species. An obvious cytopathic effect (CPE) characterized by cell shrinkage, rounding, necrosis, and detachment was observed in all cell lines except C6/36 cells (Figure 1F). Meanwhile, the NS1′ expression detected by IFA confirmed that recombinant viruses were successfully rescued in all cell lines using three types of JEV genomic RNAs, even though CPE was not observed in C6/36 cells (Figure 1F). Further, the yields of infectious virus in culture supernatants of BHK‐21, ST, and DF‐1 cells were monitored at 24, 36, and 48 h post‐transfection (hpt), and the viral titers in culture supernatants of C6/36 cells were monitored at 48, 72, and 96 hpt. Three JEV genomic RNAs produced similar viral titers in BHK‐21 and C6/36 cells at all time points (Figure 1G), which was consistent with previous studies on other flaviviruses.^[^ ^36^ ^]^ However, viral titers produced with the noncapped genomic RNA were significantly lower than those of the capped genomic RNAs in ST and DF‐1 cells (Figure 1G). These results suggested that a CI translation initiation strategy could be employed by JEV to evade host shutoff.

### 2.2 RNA Structures in UTRs Play Essential Roles in CI Translation Initiation

**Figure 2:**
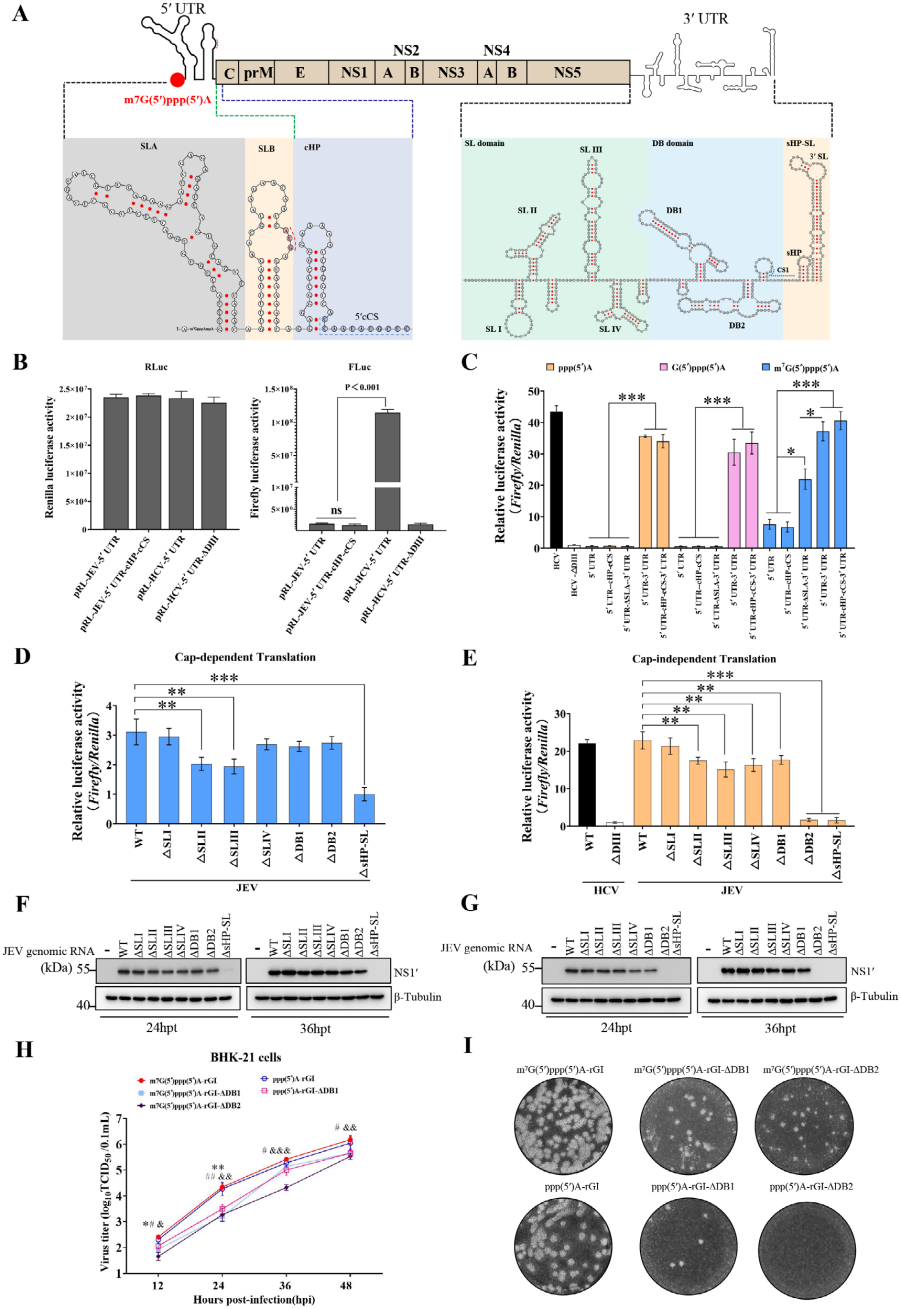
Three *cis*‐acting elements in UTRs are crucial for the CI translation initiation of JEV mRNA. A) Secondary structure diagram of the 5′‐ and 3′‐ termini of JEV. B) BHK‐21 cells were respectively transfected with bicistronic constructs pRL‐JEV‐5′UTR, pRL‐JEV‐5′UTR‐cHP‐cCS, pRL‐HCV‐5′UTR, and pRL‐HCV‐5′UTR‐ΔDIII at a dose of 2 µg. At 24 h post‐transfection, the *firefly* luciferase and *Renilla* luciferase activities in BHK‐21 cells were determined by luciferase assay (*n* = 4). C–E) Monocistronic reporter RNA or its deletion mutants were generated via T7 promoter‐mediated in vitro transcription and then co‐transfected with a 5′capped‐RLuc mRNA into BHK‐21 cells. At 12 h post‐transfection, the *firefly* and *Renilla* luciferase activity in BHK‐21 cells was determined using a dual‐luciferase reporter assay. The relative luciferase activity was calculated by normalizing *firefly* luciferase activity to *Renilla* luciferase activity (*n* = 4; *, *p *< 0.05, **, *p *< 0.01, ***, *p *< 0.001, ns, no significance; statistical significance determined by one‐way ANOVA). F,G) Western‐blot analysis of BHK‐21 cells transfected with the JEV genomic RNA with 5′termini m^7^G(5′)ppp(5′)A or ppp(5′)A of WT or the truncated. H) BHK cells were infected with viruses m^7^Gppp(5′)A‐rGI, ppp(5′)A‐rGI, m^7^G(5′)ppp(5′)A‐rGI‐ΔDB1, ppp(5′)A‐rGI‐ΔDB1 and m^7^G(5′)ppp(5′)A‐rGI‐ΔDB2 at an MOI of 0.05, respectively. At the indicated time points, culture supernatants were collected to determine virus titers by TCID_50_ assay (*n* = 3). Data are the means ± SD of three or four independent experiments, and statistical significance was tested by one‐way ANOVA analysis with Tukey's multiple comparison test. The significant differences between m^7^Gppp(5′)A‐rGI and m^7^G(5′)ppp(5′)A‐rGI‐ΔDB1 are labeled (*, *p *< 0.05; **, *p *< 0.01). The significant differences between m^7^Gppp(5′)A‐rGI and ppp(5′)A‐rGI‐ΔDB1 is marked (**^#^**, *p *< 0.05; **^##^**,*p *< 0.01). The significant differences between m^7^Gppp(5′)A‐rGI and m^7^G(5′)ppp(5′)A‐rGI‐ΔDB2 is indicated (^&^, *p *< 0.05; ^&&^, *p *< 0.01; ^&&&^, *p *< 0.001). I) Plaque morphology of the recombinant viruses in BHK‐21 cells.

In most single‐stranded positive‐sense RNA viruses without a cap structure at the 5′ end of their genomes, the IRES element situated at the 5′ end of the viral genomes is usually employed to initiate viral translation. Several secondary RNA structures at the 5′ end of the JEV genome are required for viral translation and RNA synthesis, including SLA, SLB, and cHP (**Figure** **2**A).^[^ ^38^, ^39^ ^]^ A panel of bicistronic reporter plasmids as depicted in Figure S2A (Supporting Information) was generated to evaluate the IRES or IRES‐like activity of the 5′‐end of the JEV genome. In those constructs, *Renilla* luciferase (RLuc) is expressed through CD translation, while *firefly* luciferase (Fluc) is expressed depending on the IRES or IRES‐like activity of the upstream viral sequences. HCV IRES (HCV‐5′ UTR) and inactivated HCV IRES (HCV‐5′ UTR‐ΔDIII) were used as positive and negative controls. In DNA transfected BHK‐21 cells, all constructs expressed similar high levels of RLuc, while neither 5′UTR nor 5′UTR‐cHP‐cCS effectively initiated the CI translation of FLuc in comparison to a significantly higher level of FLuc expression driven by HCV IRES (Figure 2B). To rule out the possibility that the nonviral sequence added to the 5′ terminus may interfere with the IRES activity of JEV 5′UTR, a panel of monocistronic RNA reporters without a cap structure, including JEV‐5′UTR‐FLuc, JEV 5′UTR‐cHP‐cCS‐FLuc, HCV‐5′UTR‐FLuc, and HCV‐5′UTR‐ΔDIII‐FLuc (Figure S2B, Supporting Information), were transfected into BHK‐21 cells with a 5′‐capped RLuc mRNA as an internal control. In line with results generated with the bicistronic luciferase assay, the JEV‐5′UTR‐FLuc and JEV 5′UTR‐cHP‐cCS‐FLuc mRNA produced similar low levels of FLuc as the inactivated HCV IRES, while HCV‐5′UTR‐FLuc mRNA produced a significantly higher level of FLuc (Figure 2C). Thus, the 5′UTR or 5′UTR‐cHP‐cCS of JEV does not have IRES or IRES‐like activity.

The non‐polyadenylated 3′UTR of flavivirus serves as an enhancer of viral translation initiation and translation efficiency.^[^ ^40^, ^41^ ^]^ To assess the role of JEV 3′UTR in CI translation, a panel of RNA transcripts with JEV 3′UTR following the FLuc ORF was designed (Figure S2B, Supporting Information). Three types of RNA transcripts with or without a functional cap structure were synthesized for each reporter. In RNA transfected BHK‐21 cells, all transcripts with a functional cap structure produced relatively high levels of luciferase activity, and the capped RNA transcripts with 3′UTR produced nearly fivefold more luciferase activity than the capped RNA transcripts without 3′UTR (Figure 2C), confirming that flavivirus 3′UTR contributes to the enhancement of viral translation.^[^ ^41^ ^]^ Of note, in the presence of 3′UTR, the RNA transcripts without a functional cap structure produced comparable high levels of luciferase activity as the capped RNA transcripts, implying the involvement of JEV 3′UTR in the regulation of CI translation initiation. Moreover, the absence of cHP‐cCS in JEV‐5′UTR‐FLuc‐3′UTR did not affect the expression of FLuc, suggesting that cHP and cCS are not important for CI translation (Figure 2C). Noteworthy, the essential role of the SLA structure of 5′UTR in CI translation was proved by the extremely low FLuc produced by the reporter JEV‐5′UTR‐ΔSLA‐FLuc‐3′UTR without a functional cap (Figure 2C), indicating that the integrity of JEV 5′UTR structure is also crucial for CI translation. JEV 3′UTR forms high‐order RNA structures, including SLI, SLII, SLIII, SLIV, DB1, DB2, and sHP‐SL (Figure 2A).^[^ ^8^, ^42^ ^]^ To evaluate the role of each motif of 3′UTR in CI translation, a panel of JEV‐5′UTR‐FLuc‐3′UTR mutants was generated with the individual motif deleted (Figure S2C, Supporting Information). All capped RNA transcripts effectively initiated translation in a cap‐dependent manner, although some mutants produced relatively low FLuc compared to the JEV‐5′UTR‐FLuc‐3′UTR (Figure 2D). For the CI translation using the noncapped RNA transcripts, the deletion of each motif downregulated FLuc expression and the deletion of DB2 or sHP‐SL reduced FLuc expression to background level as the negative control HCV‐5′UTR‐ΔDIII‐FLuc (Figure 2E), suggesting that DB2 and sHP‐SL are critical for JEV CI translation initiation.

Next, a panel of mutants with the individual motif of 3′UTR deleted was created with a reverse genetic system of GI JEV Beijing/2020‐1^[^ ^43^ ^]^ to confirm the crucial RNA structures of 3′UTR in the context of JEV infection. The full‐length genomic RNAs with or without a functional type I cap were respectively transfected BHK‐21 cells to rescue recombinant viruses. All RNA mutants, except for ΔsHP‐SL, successfully expressed viral protein and rescued recombinant viruses in a cap‐dependent manner (Figure 2F; Figure S3A, Supporting Information), while the noncapped RNAs of ΔDB2 and ΔsHP‐SL failed to express viral protein and rescue viruses (Figure 2G; Figure S3A, Supporting Information). These results confirmed the indispensable role of DB2 in initiating CI translation. The designed deletions of the rescued viruses (P0) were validated by Sanger sequencing (Figure S4, Supporting Information). All mutants with individual SL structure deleted exhibited comparable growth kinetics as the WT virus in BHK‐21 cells (Figure S3B,C, Supporting Information), while the mutants of ΔDB1 and ΔDB2 produced viral titers significantly lower than those of the WT virus (Figure 2H). Analysis of plaque sizes formed in BHK‐21 cells indicated that mutant viruses of ΔDB1 and ΔDB2 formed small plaques approximately twofold smaller than those formed by the WT virus (Figure 2I).

Of note, both capped and noncapped JEV genomes without sHP‐SL failed to rescue viable viruses. Considering that the cyclization sequence (3′CS) within sHP‐SL is indispensable for viral RNA synthesis and virion production,^[^ ^44^, ^45^ ^]^ we explored whether the failure of ΔsHP‐SL mutant recovery was caused by a defect of viral RNA synthesis. A JEV replicon (rGI‐GLuc) as depicted in Figure S5A (Supporting Information) allows discrimination between viral translation and viral RNA synthesis, and a replication‐defective RNA with NS5‐372‐386 deletion^[^ ^11^ ^]^ (rGI‐GLuc‐NS5mut) was included as a negative control. Viral RNA synthesis could be assessed based on the second round of GLuc accumulation for rGI‐GLuc but not for rGI‐GLuc‐NS5mut (Figure S5B, Supporting Information). The deletions of DB2 and sHP‐SL completely blocked GLuc expression in a cap‐independent manner, while the deletion of sHP‐SL also downregulated the CD translation of GLuc (Figure S5C,D, Supporting Information). Based on GLuc expression at 8–12 hpt, the replicon without sHP‐SL is replication‐defective, and the deletion of DB2 also attenuated viral RNA synthesis (Figure S5C,D, Supporting Information). Thus, DB2 and sHP‐SL within 3′UTR are essential for JEV CI translation and are also involved in viral RNA synthesis.

### 2.3 DB2 and sHP‐SL Determine JEV Evasion of Host Translational Shutoff and are Associated with the Virulence of JEV in Mice

**Figure 3:**
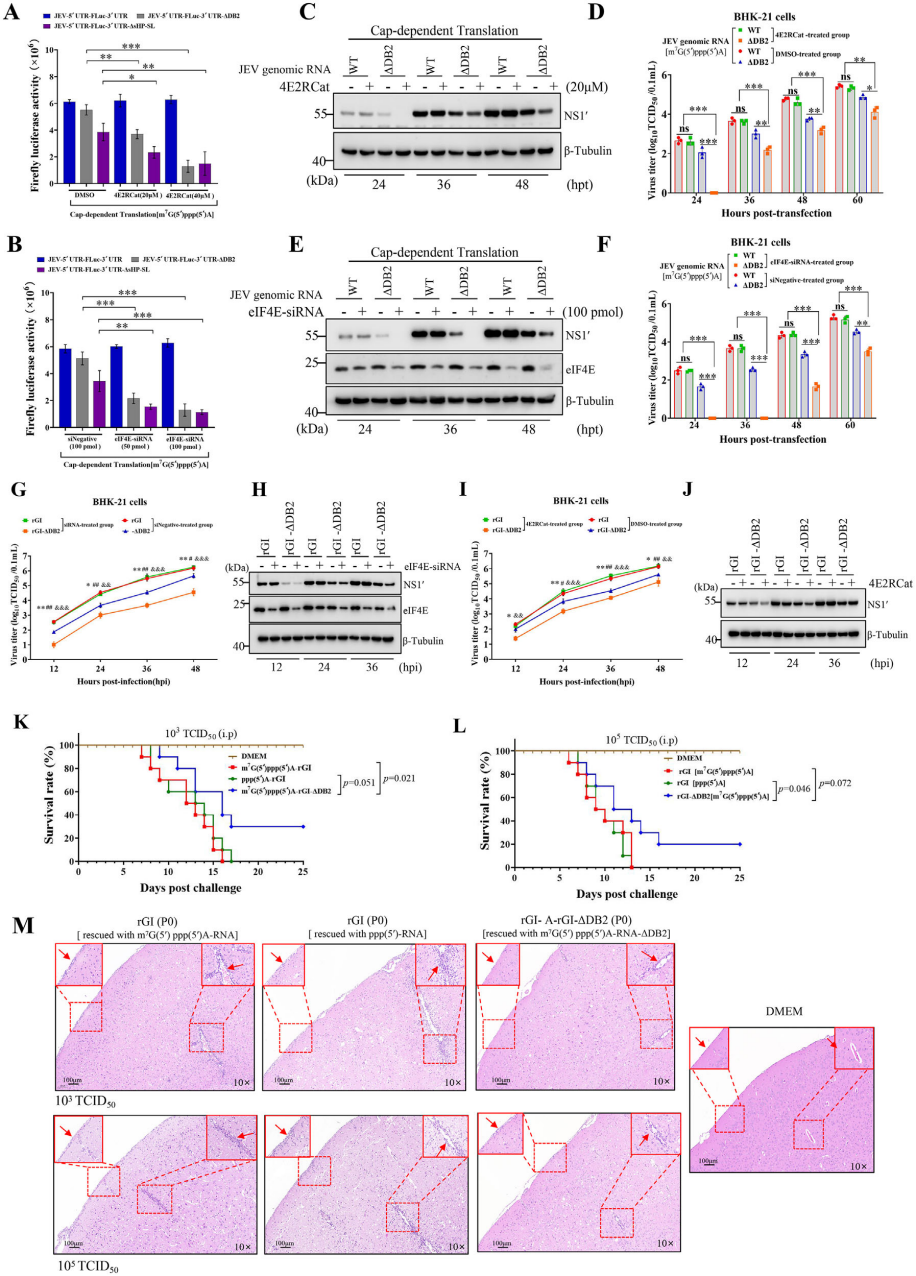
The *cis*‐acting elements DB2 and sHP‐SL of 3′UTR determine the resistance of JEV to the suppression of CD translation initiation and the virulence of JEV in mice. A,B) The *firefly* luciferase activity assay of monocistronic RNA reporters with 5′terminal modified by either m^7^G(5′)ppp(5′)A or ppp(5′) in BHK‐21 cells treated with eIF4E‐specific siRNAs or 4E2RCat (*n* = 4). C–F) BHK‐21 cells were respectively treated with eIF4E‐specific siRNAs (100 pmol) or 4E2RCat (20 µm) and then transfected with JEV genomic RNA of WT or ΔDB2 mutant with a type I cap structure. At the indicated time points of post‐transfection, NS1′ protein was detected by immunoblotting (C,E), and viral titers in culture supernatants were measured by TCID_50_ assays (D and F) (*n* = 3). G–J) BHK‐21 cells were respectively treated with eIF4E‐specific siRNAs or 4E2RCat and then infected with rGI and rGI‐ΔDB2 at an MOI of 0.05. At the indicated time points of post‐infection, viral titers in culture supernatants were measured by TCID_50_ assays (G,I) (*n* = 3), and NS1′ protein was detected by immunoblotting (H,J). K,L) The survival rate of mice (*n* = 10) intraperitoneally mock‐infected or infected with the indicated JEV at doses of 10^3^ and 10^5^ TCID_50_. The significant differences were determined by the Kaplan–Meier analysis. M) Histopathological analysis of brain lesions of the dead mice. *, *p *< 0.05; **, *p *< 0.01; ***, *p *< 0.001. Data are presented as mean ± SD and tested by one‐way ANOVA (A, B, D, F, G, and I).

The role of CI translation initiation in JEV evasion of host translational shutoff was initially studied with the monocistronic reporters in Figure S2B (Supporting Information). To validate the critical role of DB2 and sHP‐SL in JEV evasion of host translational shutoff, we first evaluated the translational activity of luciferase reporter RNA in the presence of suppression of CD translation. The suppression of CD translation initiation significantly reduced FLuc expression of reporter RNAs without DB2 or sHP‐SL in a dose‐dependent manner but not that of WT (**Figure** **3**A,B). Furthermore, the suppression of CD translation resulted in a decrease in the translational efficiency of viral RNA without DB2 or sHP‐SL (Figure 3C,E; Figure S6, Supporting Information), and extremely low viral titers with the capped ΔDB2 RNA at all time points (Figure 3D,F), suggesting that both DB2 and sHP‐SL are critical for JEV evasion of the shutoff of CD translation. Meanwhile, the effect of suppressing CD translation initiation on the growth kinetics of rGI and rGI‐ΔDB2 was further evaluated. Similar growth kinetics of rGI were observed in BHK‐21 cells with/without CD translation attenuated (Figure 3G,I). In contrast, eIF4E knockdown and 4E2RCat treatment significantly attenuated the growth of rGI‐ΔDB2 (Figure 3G,I). Consistently, the expression of NS1′ was downregulated by eIF4E knockdown and 4E2RCat treatment for rGI‐ΔDB2 but not WT virus (Figure 3H,J). Thus, DB2 and sHP‐SL are critical for JEV evasion of host translational shutoff.

Next, the effect of DB2 deletion on the pathogenicity of JEV was evaluated via mice challenge. All mice succumbed to challenges at different doses in the rGI groups, while 20%–30% survival rates were observed in the rGI‐ΔDB2 groups (Figure 3K,L). In the brains of dead mice, viral RNA was detected by RT‐PCR, and the DB2 deletion was verified. The characteristic lesion of JEV encephalitis was observed in the brain of all dead mice, but it was reduced in the rGI‐ΔDB2 group (Figure 3M). These results suggested that DB2 is associated with the virulence of JEV in mice. Due to the lethal effect of sHP‐SL deletion on JEV, it is not possible to assess the role played by sHP‐SL in JEV pathogenicity.

### 2.4 The Critical Role of DB2 and sHP‐SL in the CI Translation is Highly Conserved in the Genus *orthoflavivirus*

**Figure 4:**
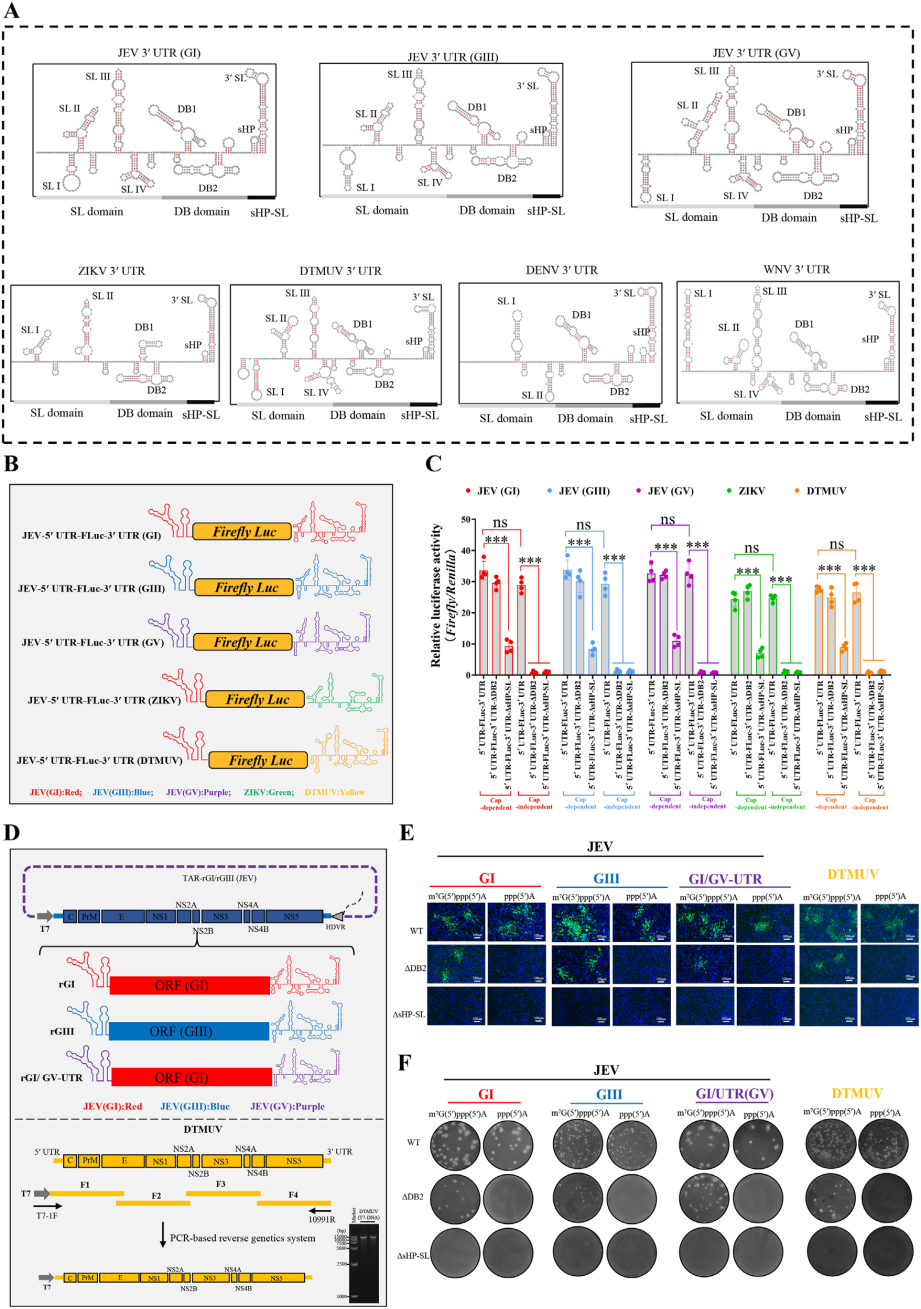
The critical role of DB2 and sHP‐SL within 3′UTR in CI translation initiation is evolutionarily conserved in various flaviviruses. A) Secondary structure diagram of 3′UTR of various genotypes JEV, ZIKV, DENV, WNV and DTMUV. B) Diagrams of flaviviruses monocistronic reporter constructs controlled by a T7 promoter: JEV‐5′UTR‐FLuc‐3′UTR(GI), JEV‐5′UTR‐FLuc‐3′UTR (GIII), JEV‐5′UTR‐FLuc‐3′UTR (GV), ZIKV‐5′ UTR‐FLuc‐3′ UTR and DTMUV‐5′UTR‐FLuc‐3′UTR. C) Monocistronic RNA reporters with 5′terminal modified by either m^7^G(5′)ppp(5′)A or ppp(5′) were generated via T7 promoter‐mediated in vitro transcription, and then co‐transfected with a 5′‐capped‐RLuc mRNA into BHK‐21 cells. At 12 h post‐transfection, the cell samples were harvested for *firefly* and *Renilla* luciferase activity assay (*n* = 4). The relative luciferase activity was calculated by normalizing *firefly* luciferase activity to *Renilla* luciferase activity. ***, *p* < 0.001; **, *p* < 0.01; ns, no significance, tested by the one‐way ANOVA analysis with Tukey's multiple comparison test(C). Data are the means ± SD of the results of four independent experiments. D) Schematic illustration of constructed infectious clones of GI JEV, GIII JEV, and GI/GV‐UTR JEV, and a PCR‐based reverse genetics system of DTMUV. E) Immunofluorescence analysis of recombinant viruses infection in BHK‐21 cells. F) Plaque morphology of recombinant viruses in BHK‐21 cells.

CI translation initiation has been proven in several flaviviruses.^[^ ^33^, ^35^, ^36^ ^]^ Based on in silico prediction, the secondary structures of DB2 and sHP‐SL are highly conserved among different JEV genotypes, ZIKV, WNV, DENV, and DTMUV (**Figure** **4**A). We speculated that the function of DB2 and sHP‐SL in regulating CI translation is conserved in flaviviruses. A panel of monocistronic FLuc reporters carrying different flavivirus UTRs with/without the deletion of DB2 or sHP‐SL was utilized to evaluate the function of flavivirus UTRs in the CI translation (Figure 4B). As expected, all noncapped reporter RNAs flanked by 5′UTR of JEV and 3′UTR of ZIKV, DTMUV, or various genotypes of JEV produced comparable levels of FLuc as the corresponding capped RNAs (Figure 4C). Noteworthy, all reporter RNAs with the absence of either DB2 or sHP‐SL produced extremely low levels of FLuc, suggesting that DB2 and sHP‐SL are critical for CI translation initiation in various genotypes of JEV and other flaviviruses. We also confirmed these results in the context of virus recovery of GIII and GV JEV as well as DTMUV. As illustrated in Figure 4D, infectious cDNA clones of GIII and chimeric GI/GV‐UTR, and the PCR‐based reverse genetics system for DTMUV^[^ ^46^ ^]^ were used to create mutants with a deletion of DB2 or sHP‐SL. The capped and noncapped genomic RNAs were transfected into BHK‐21 cells to rescue recombinant viruses. All WT genomes produced infectious viruses, while the absence of DB2 and sHP‐SL prevented three JEV genotypes and DTMUV from the CI translation leading to the failure of virus recovery (Figure 4E,F). These results further supported that DB2 and sHP‐SL are essential to the CI translation in various genotypes of JEV as well as DTMUV. Taken together, the decisive role of DB2 and sHP‐SL in regulating CI translation is evolutionarily conserved in various flaviviruses.

### 2.5 Cellular DDX3 and PABP1 are Involved in JEV CI Translation

**Figure 5:**
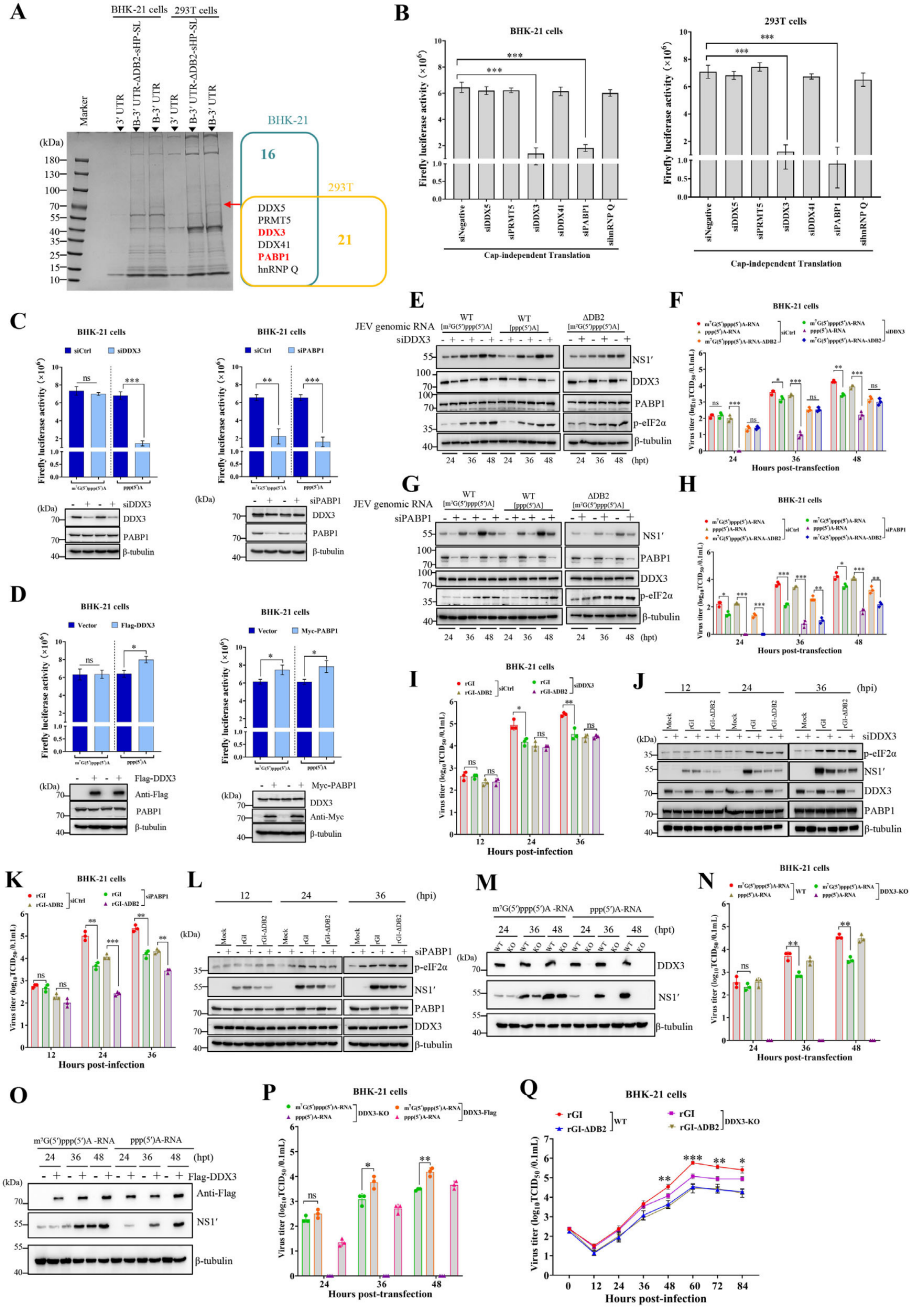
DDX3 and PABP1 regulate CI translation initiation of JEV in vitro. A) Identification of cellular proteins associated with JEV 3′UTR and JEV 3′UTR‐ΔDB2‐sHP‐SL (left). Venn diagram of DB2 and sHP‐SL specific binding proteins in BHK‐21 and 293T cells detected by RNA pull‐down and mass spectrometry (right). B) The *firefly* luciferase activity assay using the noncapped monocistronic RNA reporter JEV‐5′UTR‐FLuc‐3′UTR in BHK‐21 and 293T cells with an indicated protein silenced using siRNAs (*n* = 3). C) BHK‐21 cells pre‐treated with 100 pmol of a mixture of DDX3‐specific siRNAs (left) or PABP1‐specific siRNAs (right) were respectively transfected with monocistronic RNA reporters JEV 5′ UTR‐FLuc‐3′ UTR with 5′terminal modified by either m^7^G(5′)ppp(5′)A or ppp(5′), and then harvested at 12 h post‐transfection for analysis of *firefly* luciferase activities (*n* = 4). The levels of endogenous DDX3 and PABP1 were detected by western blotting. D) The *firefly* luciferase activity assay of monocistronic RNA reporter JEV‐5′UTR‐FLuc‐3′UTR in BHK‐21 cells with Flag‐DDX3 (left) or Myc‐PABP1 (right) over‐expressed (*n* = 4). E–H) BHK‐21 cells with DDX3 or PABP1 silenced were transfected with the capped JEV genomic RNA of WT or ΔDB2 mutant, or noncapped WT genomic RNA. At the indicated time points post‐transfection, the expression of viral NS1′ protein, p‐eIF2α, DDX3, and PABP1 in BHK‐21 cells was analyzed by immunoblotting (E,G), and viral titers in culture supernatants were determined by TCID_50_ assays (F and H) (*n* = 3). I–L) BHK‐21 cells with DDX3 or PABP1 silenced were infected with rGI and rGI‐ΔDB2 at a dose of 0.05 MOI. Replication of rGI and rGI‐ΔDB2 was monitored by TCID_50_ assays (I and K) (*n *= 3). The expression of viral NS1′ protein, p‐eIF2α, DDX3, and PABP1 in BHK‐21 cells was analyzed by immunoblotting (J,L). M–P) The translational activity of JEV genomic RNA in DDX3‐KO BHK‐21 cells. DDX3‐KO cells with or without DDX3‐Flag over‐expression were transfected with JEV genomic RNA m^7^G(5′)ppp(5′)A‐RNA, ppp(5′)A‐RNA, respectively. At different time points post‐transfection, immunoblotting analysis of viral NS1′ protein (M,O). Virus titers in culture supernatant were evaluated by TCID_50_ assays (N and P) (*n* = 3). Q) The replication ability of rGI and rGI‐ΔDB2 in WT and DDX3‐KO BHK‐21 cells (*n* = 3). The significant differences between WT and DDX3‐KO BHK‐21 cells are marked (*, *p *< 0.05; **, *p *< 0.01; **, *p *< 0.001). *, *p *< 0.05; **, *p *< 0.01; ***, *p *< 0.001; ns, no statistical differences. Data are presented as mean ± SD of three independent experiments (B, C, D, F, H, I, K, N, P, and Q).

As obligate intracellular parasites, viruses must exploit the host translation apparatus to support viral protein synthesis. The interactions between viral UTR and cellular factors are believed to be the first step of viral translation initiation. A biotinylated RNA pull‐down assay was performed to identify host proteins associated with DB2 and sHP‐SL of JEV. As shown in **Figure** **5**A, a unique band specifically associated with the biotinylated JEV 3′UTR instead of biotinylated JEV 3′UTR‐ΔDB2‐sHP‐SL was observed for two cell lines. LC‐MS/MS analysis with this band identified 16 proteins for BHK‐21 cells and 21 proteins for 293T cells, with six proteins shared by the two cell lines (Figure 5A; Figure S7, Table S1, Supporting Information). The shared six proteins were respectively knocked down using siRNA in BHK‐21 and HEK‐293T cells to test whether they play roles in CI translation. The cap‐independent FLuc translation of JEV‐5′UTR‐FLuc‐3′UTR was significantly reduced only when DDX3 or PABP1 was knocked down (Figure 5B). The siRNA knockdown of DDX3 did not affect the cap‐dependent FLuc expression, while PABP1 knockdown reduced 60% of CD translation (Figure 5C). Consistently, the overexpression of DDX3 specifically enhanced cap‐independent FLuc expression, while the ectopically expressed PABP1 promoted both cap‐dependent and cap‐independent FLuc expression (Figure 5D). To further strengthen the authentic roles of DDX3 and PABP1 in regulating CI translation of JEV, the translational activity of viral genomic RNA was tested in DDX3 or PABP1 knock‐down BHK‐21 cells. The siRNA knockdown of DDX3 significantly reduced NS1′ protein expression and viral production of the noncapped WT JEV genome (Figure 5E,F). Besides downregulating the translational activity of the noncapped WT RNA, DDX3 knockdown also obviously weakened NS1′ protein expression and viral production of capped WT RNA, but did not affect those of capped ΔDB2 mutant RNA that is defective in CI translation (Figure 5E,F). Of note, PABP1 knockdown reduced both the CD translation of WT RNA and ΔDB2 mutant RNA and the CI translation of WT RNA (Figure 5G,H), which is consistent with the results using FLuc reporters (Figure 5C), indicating that PABP1 is required for both CD and CI translations of JEV mRNA. Thus, we confirmed that both cellular DDX3 and PABP1 are involved in JEV CI translation.

The function of DDX3 and PABP1 in JEV replication was further evaluated. The siRNA‐mediated knockdown of DDX3 expression dramatically attenuated rGI replication by tenfold to 100‐fold at 24 and 36 hpi but did not affect the replication of rGI‐ΔDB2 that is defective in CI translation (Figure 5I,J). Besides downregulating the titers of rGI at 24 and 36 hpi, PABP1 knockdown also exhibited an inhibitory effect on the replication of rGI‐ΔDB2 (Figure 5K,L). Meanwhile, we generated a DDX3‐KO cell strain by editing the DDX3 gene but failed to obtain PABP1‐KO cells. The expression of DDX3 in DDX3‐KO cells was completely knocked out by a deletion of five nucleotides in exon 1 (Figure S8A–C, Supporting Information). The knockout of DDX3 did not affect cell proliferation (Figure S8D, Supporting Information). Virus recovery of rGI was attempted using WT and DDX3‐KO BHK‐21 cells. Only the transfection of DDX3‐KO cells with the noncapped genomic RNA failed to rescue viable viruses, and viral titers rescued with the capped RNA in DDX3‐KO cells were decreased (Figure 5M,N). Consistently, ectopically expressed DDX3‐Flag in DDX3‐KO cells rescued rGI with noncapped RNA and increased virus titers rescued with capped RNA (Figure 5O,P). Based on viral growth curves, rGI growth in DDX3‐KO cells was significantly alleviated since 48 hpi compared to that in WT cells, while no obvious difference was observed in virus titers of rGI‐ΔDB2 between WT and DDX3‐KO cells (Figure 5Q). Taken together, the loss of function and gain of function experiments demonstrated that DDX3 and PABP1 are host factors positively regulating CI translation of JEV, and PABP1 is also important for CD translation.

### 2.6 The RNA Structures of UTRs Bound by DDX3 and PABP1 Determine the CI Translation of JEV

**Figure 6:**
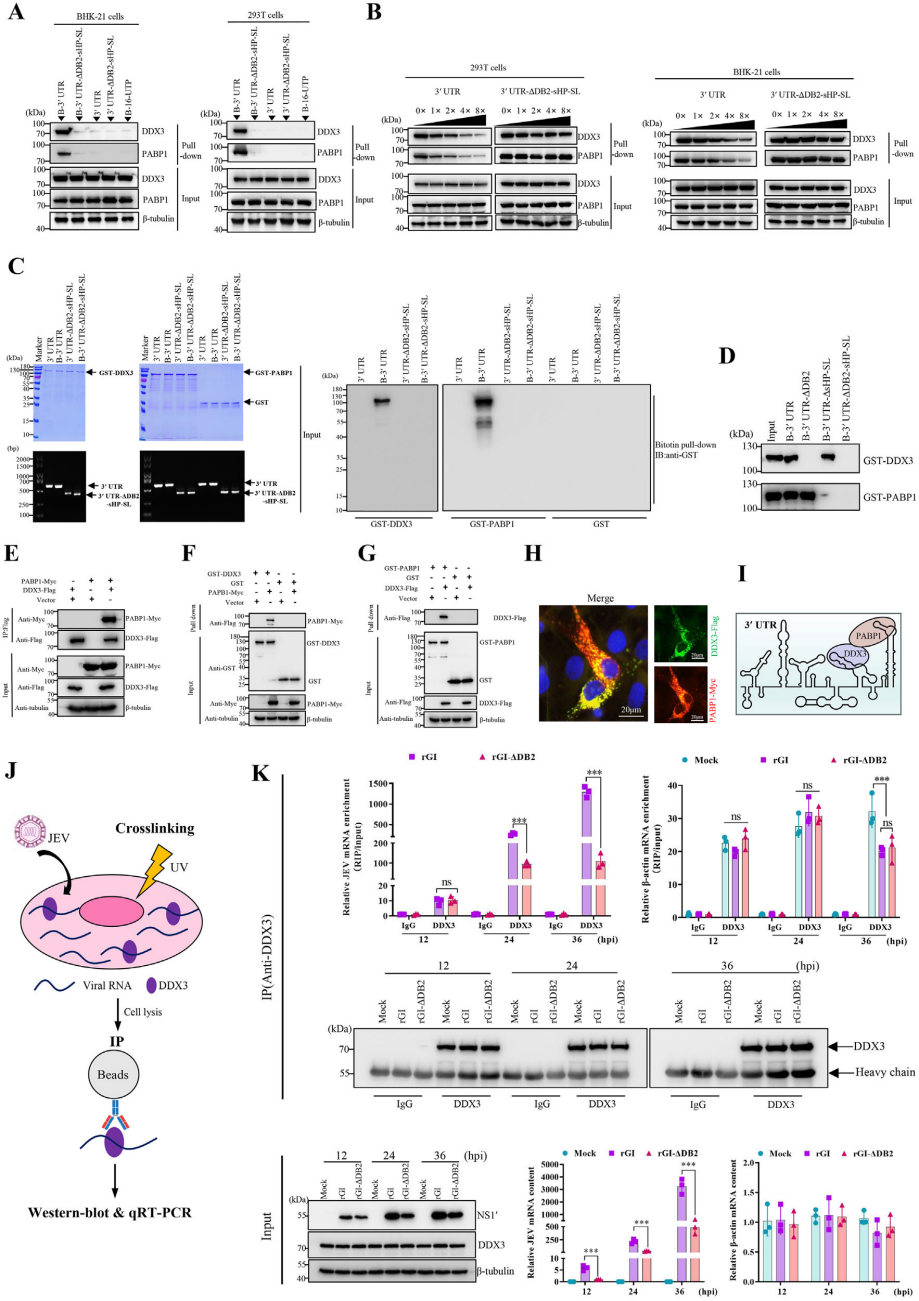
The interactions among DDX3, PABP1, and JEV 3′UTR. A) The cell lysates of BHK‐21 and 293T cells were respectively incubated with biotinylated‐3′UTR (B‐3′UTR), biotinylated‐3′UTR‐ΔDB2‐sHP‐SL (B‐3′UTR‐ΔDB2‐sHP‐SL), nonbiotinylated‐3′UTR (3′UTR) or nonbiotinylated‐3′UTR‐ΔDB2‐sHP‐SL(3′UTR‐ΔDB2‐sHP‐SL). The bound complexes were analyzed by immunoblotting. B) The interactions between DDX3/PABP1 and JEV 3′UTR were confirmed by competition assays. C,D) RNA pulldown analysis of the interaction between nonbiotinylated or biotinylated 3′UTR and recombinant proteins GST‐DDX3, GST‐PABP1, or GST. The bound complexes were analyzed by immunoblotting, and recombinant proteins and RNA in the input were detected by Coomassie brilliant blue staining and RT‐PCR. E) Co‐immunoprecipitation analysis of the interaction between the ectopically expressed DDX3‐Flag and Myc‐PABP1 in HEK293T cells. F,G) GST pulldown analysis of the interaction between recombinant DDX3 and PABP1. H) Immunofluorescent analysis of the interaction between DDX3‐Flag and PABP1‐Myc in BHK‐21 cells. I) A model for the interactions among DDX3, PABP1, and JEV 3′UTR. J) Schematic diagram of the RIP assay. BHK‐21 cells are infected with rGI or rGI‐ΔDB2 at an MOI of 0.01. At 12, 24, and 36 h post‐infection, the cells are cross‐linked with short‐wave UV light to form RNA‐protein complexes and then lysed for RNA immunoprecipitation. K) Top panel: RIP‐qPCR analysis quantifying the immunoprecipitation efficiency of JEV and β‐actin mRNA using either anti‐IgG or anti‐DDX3 antibody‐conjugated beads. Results are presented as fold‐change values normalized to input samples, with the IgG control group set as 1 for comparative analysis. Bottom panel: Quantitative analysis showing: (Left) intracellular DDX3 protein expression levels by Western blot; (Middle) JEV mRNA and (Right) β‐actin mRNA levels by RT‐qPCR. *, *p *< 0.05; **, *p *< 0.01; ***, *p *< 0.001; ns, no statistical differences. Data are presented as mean ± SD of three independent experiments.

An RNA pull‐down assay was conducted to verify the interactions among DDX3, PABP1, and JEV 3′UTR. DDX3 and PABP1 were pulled down by biotinylated 3′UTR but not by biotinylated 3′UTR‐ΔDB2‐sHP‐SL (**Figure** **6**A), suggesting that DB2 and sHP‐SL bridge the interactions between DDX3/PABP1 and JEV 3′UTR. In a competition test, the interactions DDX3/PABP1 with JEV 3′UTR were significantly out‐competed by nonbiotinylated JEV 3′UTR in a dose‐dependent manner, but not by JEV 3′UTR‐ΔDB2‐sHP‐SL (Figure 6B). To exclude the interference of other host factors, purified GST‐DDX3 and GST‐PABP1 were used for RNA pull‐down assay. GST‐DDX3 and GST‐PABP1, but not GST, coprecipitated with biotin‐labeled JEV 3′UTR in vitro (Figure 6C), indicating that DDX3 and PABP1 directly bind to JEV 3′UTR. Moreover, DDX3 was pulled down by biotinylated 3′UTR and 3′UTR‐ΔsHP‐SL, while PABP1 was pulled down by biotinylated 3′UTR and 3′UTR‐ΔDB2 (Figure 6D). These results demonstrated two pairs of direct interactions, DDX3/DB2 and PABP1/sHP‐SL. In addition, the interaction between DDX3 and PABP1 was confirmed using co‐IP (Figure 6E), GST pull‐down (Figure 6F,G), and confocal microscopy (Figure 6H). Taken together, the interactions (Figure 6I) among DDX3, PABP1, and JEV 3′UTR (DB2 and sHP‐SL) could be essential for JEV CI translation.

To examine the interaction between DDX3 and viral RNA in infected cells, we infected BHK‐21 cells with either the WT virus rGI or the mutant virus rGI‐ΔDB2. At different time points post‐infection, cells were cross‐linked with short‐wave UV light to fix cellular RNA‐protein complexes for RNA immunoprecipitation using a DDX3 antibody or an IgG control (Figure 6J). The enrichment of JEV RNA in the RNA‐protein complex immunoprecipitated by the DDX3 antibody was analyzed by RT‐qPCR. As shown in Figure 6K, in comparison with the IgG control, the DDX3 antibody pulled down significantly higher levels of JEV mRNA. In the RNA‐protein complex immunoprecipitated by the DDX3 antibody, the levels of rGI‐ΔDB2 genomic RNA were significantly lower than those of the WT genomic RNA at 24 and 36 hpi. These results confirmed that DDX3 binds viral RNA in JEV‐infected cells and DB2 of 3′UTR plays a key role in this interaction.

**Figure 7:**
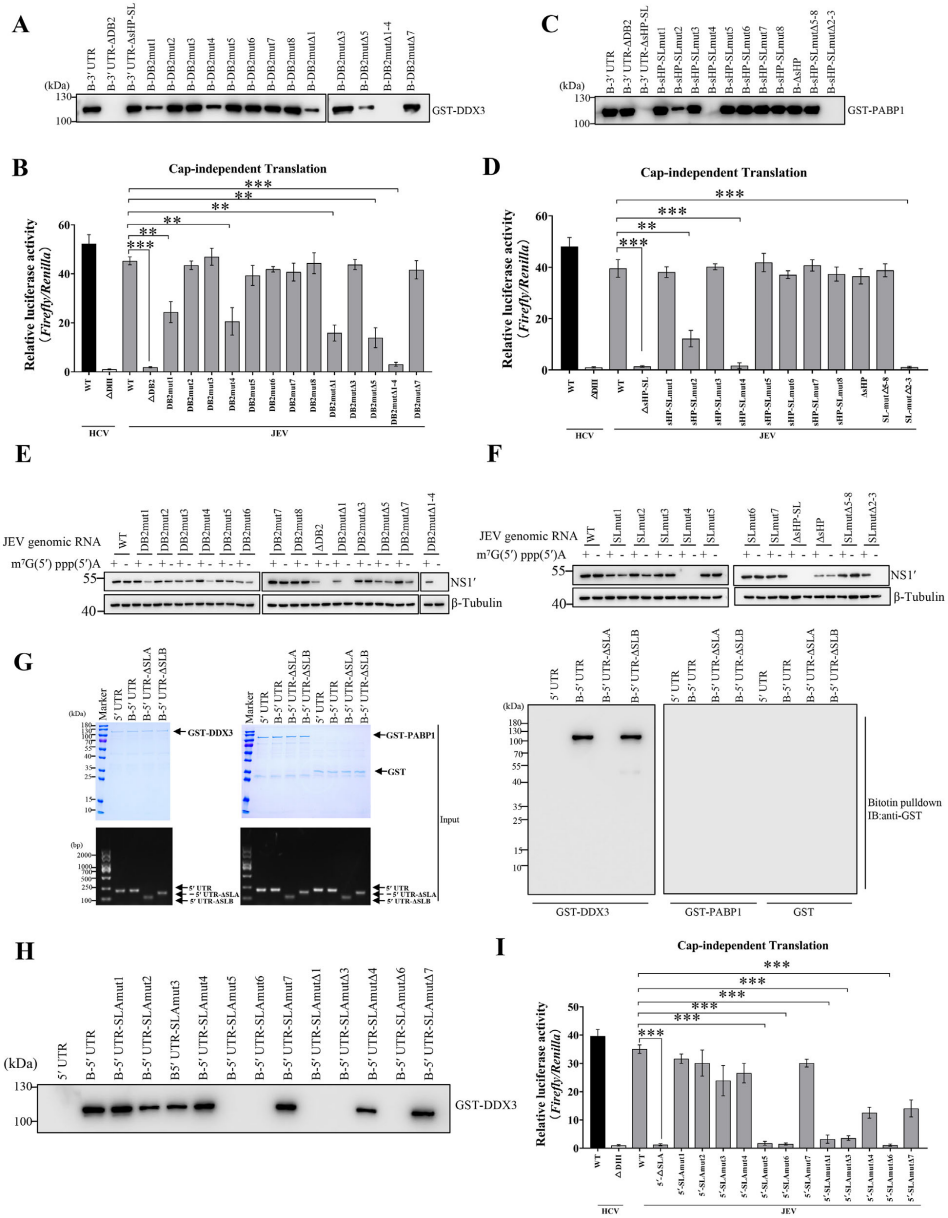
The key RNA structures in UTRs bound by DDX3 and PABP1 are critical to the CD translation initiation of JEV. A,C) Mapping interaction regions in DB2 and sHP‐SL RNA structures for DDX3 and PABP1. Pulldown assays were performed with biotinylated‐JEV 3′UTR or 3′UTR mutants and the purified recombinant GST‐DDX3(A) or GST‐PABP1 (C). The bound complexes were analyzed by immunoblotting. B,D,I) The relative luciferase activity assay of JEV‐RLuc reporter mutants in BHK‐21 cells. BHK‐21 cells were co‐transfected with the indicated noncapped reporters and a 5′‐capped‐RLuc mRNA and then lysed at 12 h post‐transfection for analyzing the luciferase activities (*n* = 4). The relative luciferase activity was calculated by normalizing *firefly* luciferase activity to *Renilla* luciferase activity. E,F) Western‐blot analysis of BHK‐21 cells transfected with the capped and noncapped viral genomic RNAs carrying the corresponding DB2 and sHP‐SL mutations or deletions. G) RNA pulldown analysis of the interaction between nonbiotinylated or biotinylated 5′UTR and recombinant GST‐DDX3, GST‐PABP1, or GST. The bound complexes were analyzed by immunoblotting, and recombinant proteins and RNA in the input were detected by Coomassie brilliant blue staining and RT‐PCR. H) Mapping regions in 5′UTR bound by DDX3. **, *p* < 0.01; ***, *p* < 0.001. Data are presented as mean ± SD from four independent experiments (B, D, and I).

To pinpoint the key RNA structures in DB2 and sHP‐SL mediated their interactions with DDX3 and PABP1, a panel of JEV 3′UTR mutants was generated by introducing mutations or deletions (Figure S9A, Supporting Information). All mutants except ΔDB2 and DB2mutΔ1‐4 bind to DDX3, and the binding of DDX3 to DB2mut1, DB2mut4, DB2mutΔ1, and DB2mutΔ5 was reduced (**Figure** **7**A). Using the monocistronic reporters containing the corresponding mutations at DB2, the disrupted RNA structures in DB2 by DB2mut1 and DB2mut4 consistently decreased the cap‐independent FLuc expression (Figure 7B). Meanwhile, the capped and noncapped viral genomic RNAs with the corresponding DB2 mutations or deletions were used to rescue viruses. Recombinant viruses were successfully recovered using all RNA transcripts except for noncapped‐ΔDB2, DB2 mutΔ1, and DB2mutΔ1‐4 which are unable to bind to DDX3 (Figure 7E, Table S2, Supporting Information). Thus, the disrupted RNA structures in DB2 by DB2mut1 and DB2mut4 mediate the interaction between DB2 and DDX3, which is also essential for the CI translation of JEV. Meanwhile, using the JEV 3′UTR mutants with modified sHP‐SL (Figure S9B, Supporting Information), the loop1 and loop2 structures of SL disrupted by mut4 and SL mutΔ2‐3 was identified as the key region that mediated the interaction between 3′UTR and PABP1 (Figure 7C). Consistently, this key structure is also critical for the cap‐independent FLuc expression in the reporter assay (Figure 7D). The corresponding mutations or deletions in sHP‐SL were also introduced into the viral genome to rescue viruses. We did not detect the viral NS1′ protein expression and failed to rescue viruses with ΔsHP‐SL and SLmut4 mutants (Figure 7F, Table S2, Supporting Information). Of note, unlike the ΔsHP‐SL mutant, the SLmutΔ2‐3 mutant was rescued using capped viral genomic RNA but not the noncapped one (Table S2, Supporting Information). Therefore, we eventually identified the key RNA structures in DB2 and sHP‐SL of 3′UTR which regulate JEV CI translation through their interactions with cellular DDX3 and PABP1.

Previous studies have revealed that DDX3 binds to both UTRs of the JEV genome to regulate viral translation.^[^ ^19^ ^]^ We further explored whether DDX3 or PABP1 binds to 5′UTR to regulate JEV CI translation. In line with a previous study,^[^ ^19^ ^]^ DDX3, instead of PABP1, directly binds to the SLA structure of 5′UTR in the RNA pull‐down assay (Figure 7G). A panel of 5′UTR mutants was generated to narrow down the key RNA structures in SLA mediating the interaction between 5′UTR and DDX3 (Figure S9B, Supporting Information). The GST pull‐down results revealed that DDX3 interacted directly with the stem‐loop1 and stem‐loop2 structures of SLA because the modifications of this structure reduced or diminished the interaction (Figure 7H). In the CI translation assay, the 5′UTR mutants that can't interact with DDX3 are defective in FLuc expression (Figure 7I), confirming the involvement of 5′UTR in JEV CI translation through its interaction with DDX3.

### 2.7 DDX3 Does Not Rely on its RNA Helicase Activity to Regulate JEV CI Translation

**Figure 8:**
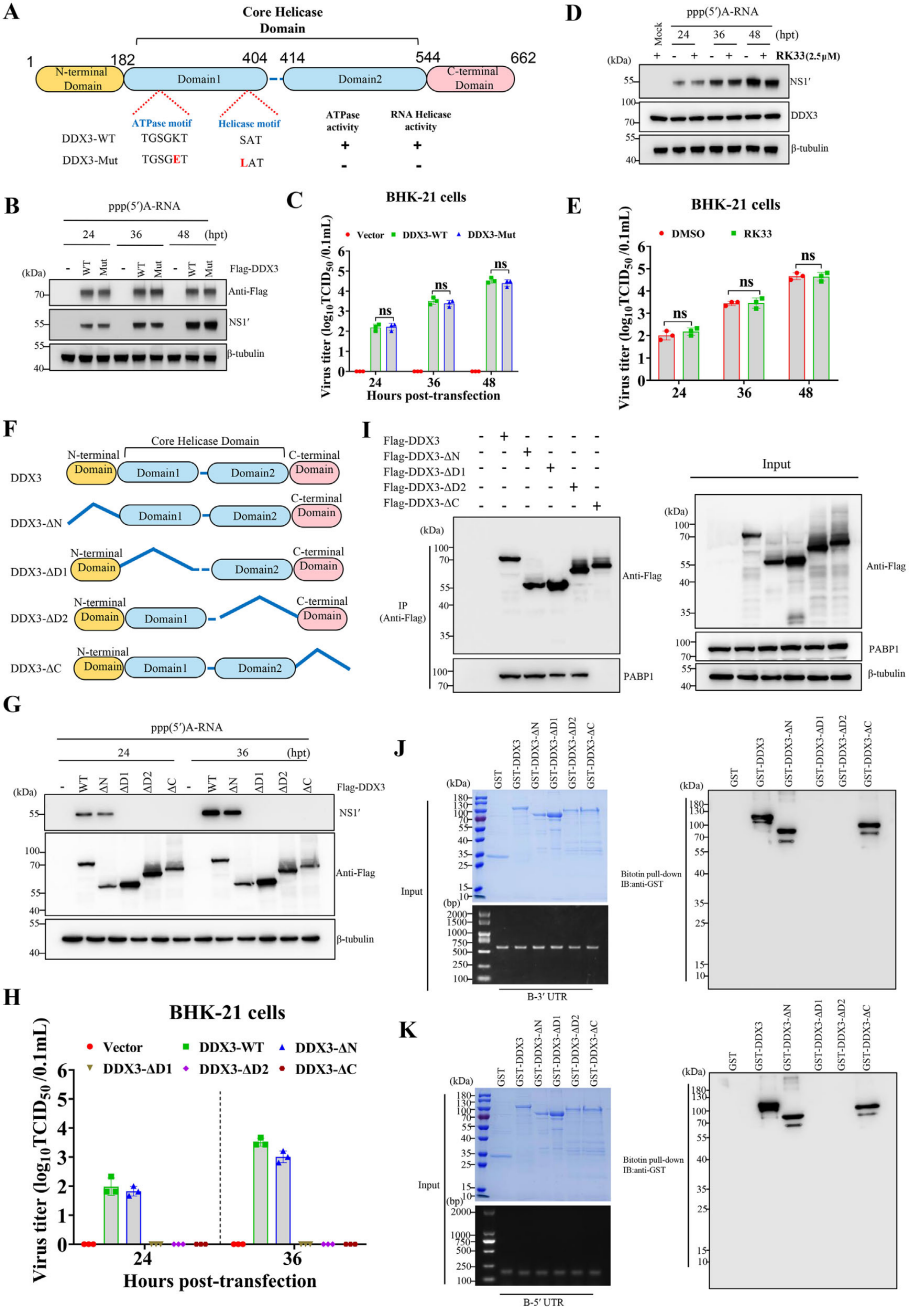
The regulation of JEV CI translation by DDX3 relies on its interaction with JEV UTRs and PABP1 but not its helicase activity. A) Schematic representation of the WT and mutant DDX3. The lysine (K) in the ATPase motif and the serine (S) in the helicase motif were respectively changed into glutamic acid (E) and leucine (L) in DDX3‐Mut. +, presence of ATPase or RNA helicase activities; ‐, absence of ATPase or RNA helicase activities. B,C) The translational activity of JEV genomic RNA in DDX3‐KO BHK‐21 cells. DDX3‐KO cells with DDX3‐WT‐Flag or DDX3‐Mut‐Flag overexpression were transfected with noncapped JEV genomic RNA. At different time points post‐transfection, immunoblotting analysis of viral NS1′ protein (B). Virus titers in culture supernatants were evaluated by TCID_50_ assays (C) (*n* = 3). D,E) BHK‐21 cells were treated with RK‐33 (2.5 µm) and then transfected with noncapped JEV genomic RNA of WT. At indicated time points post‐transfection, NS1′ protein was detected by immunoblotting (D), and viral titers in culture supernatants were determined by TCID_50_ assays (E) (*n* = 3). F) Schematic representation of the truncated mutants of DDX3. G,H) The translational activity of JEV genomic RNA in DDX3‐KO BHK‐21 cells with the overexpression of DDX3 or DDX3 mutants. I) Co‐immunoprecipitation analysis of the interaction between the ectopically expressed DDX3‐Flag or its mutants and Myc‐PABP1 in HEK‐293T cells. J,K) RNA pulldown analysis of the interaction between biotinylated 5′UTR or 3′UTR and recombinant GST‐DDX3, GST‐DDX3‐ΔN, GST‐DDX3‐ΔD1, GST‐DDX3‐ΔD2, GST‐DDX3‐ΔC or GST. Data are presented as mean ± SD of three independent experiments (C, E, and H). **, *p* < 0.01; ***, *p* < 0.001; ns, no statistical difference.

Since DDX3 belongs to the DEAD box RNA helicase family that harbors ATPase and RNA helicase activities,^[^ ^47^ ^]^ we sought to determine whether the RNA helicase activity of DDX3 is required for JEV CI translation. The DDX3‐Mut containing mutations at the ATPase motif (K to E) and helicase motif (S to L) of DDX3 was constructed to eliminate its RNA helicase activity as described previously^[^ ^48^, ^49^ ^]^ (**Figure** **8A**), and the fluorescence resonance energy transfer assay was used to analyze DDX3‐Mut helicase activity. As shown in Figure S10 (Supporting Information). the mutations at the ATPase motif (K to E) and helicase motif (S to L) of DDX3 successfully eliminated its RNA helicase activity. Further, the recovery of rGI was attempted using DDX3‐KO cells with or without the overexpression of DDX3 or DDX3‐Mut. Recombinant rGI was rescued using noncapped RNA transcript with the help of DDX3‐WT‐Flag or DDX3‐Mut‐Flag, while virus recovery failed in DDX3‐KO cells (Figure 8B,C). Meanwhile, the downregulation of DDX3 helicase activity by the specific inhibitor RK33^[^ ^50^ ^]^ in BHK‐21 cells, had no obvious effect on NS1′ expression and viral yields (Figure 8D,E), suggesting that DDX3 does not rely on its RNA helicase activity to regulate JEV CI translation.

DDX3 contains a highly variable N‐terminal domain core‐helicase domain, a core‐helicase domain that is composed of two Rec‐like domains (D1/2) in tandem and a C‐terminal domain (Figure 8A). To determine which domain of DDX3 is essential for JEV CI translation, we constructed several DDX3 mutants with deletions and assessed their function in DDX3‐KO cells (Figure 8F). We found that the deletion of any domains other than the N‐terminal domain led to the loss of viral protein expression and the failure of virus recovery using noncapped viral RNA transcript in DDX3‐KO cells (Figure 8G,H). Furthermore, the interaction between DDX3 and PABP1 was disrupted by the C‐terminal domain deletion of DDX3 (Figure 8I), and the absence of either helicase domain resulted in the loss of the ability of DDX3 to bind to the 5′/3′UTRs of JEV (Figure 8J,K). Thus, the C‐terminal domain and helicase domains of DDX3 respectively mediate its interactions with PABP1 and JEV UTRs, which are critical for the CI translation of JEV.

### 2.8 DDX3 Interacts with PABP1 to Form a Translation Initiation Complex for Recruiting 43S PIC to JEV 5′UTR

**Figure 9:**
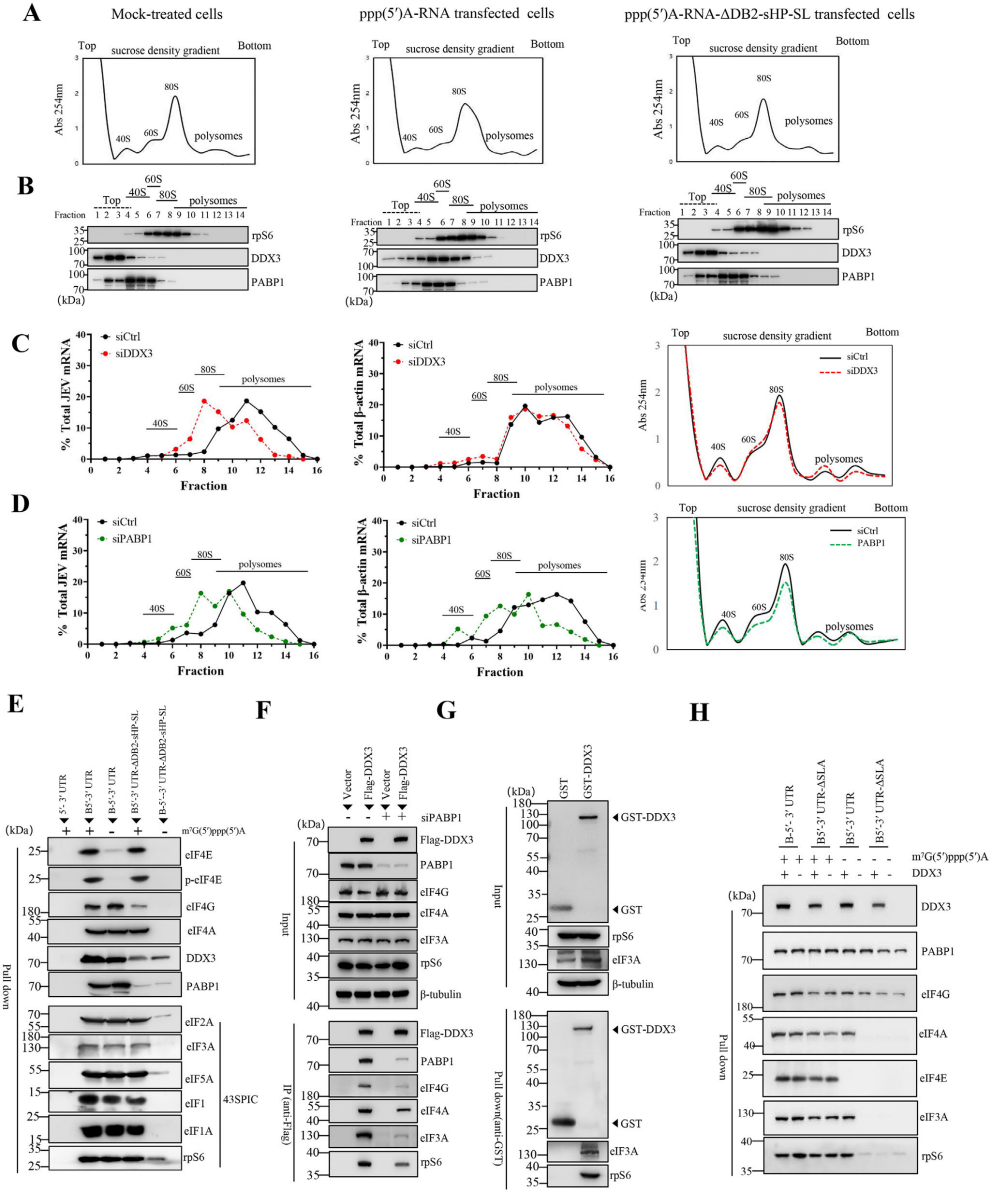
DDX3 cooperates with PABP1 to recruit translation initiation factors and 43S PIC for optimal CI translation initiation of JEV. A,B) Ribosome profiles of BHK‐21 cells mock‐transfected or transfected with noncapped JEV genome or ΔDB2‐sHP‐SL mutant. The ribosome profiles were obtained by measuring the absorbance at 254 nm of individual fractions (A). Each fraction was immunoblotted using the antibodies rpS6, DDX3, and PABP1 (B). C,D) BHK‐21 cells treated with either a control siRNA (siCtrl) or siRNA targeting DDX3 (C) or PABP1 (D) were transfected with JEV ppp(5′)A‐RNA and ppp(5′)A‐RNA‐ΔDB2‐sHP‐SL at a dose of 2 µg. At 8 h post‐transfection, cell lysates were resolved and fractionated through sucrose gradients. Each fraction was subjected to RT‐qPCR analysis of JEV and β‐actin mRNA levels. The percentage of mRNA transcripts recovered from each fraction was plotted against the fraction number. E,H) RNA pulldown analysis of host factors bound by nonbiotinylated or biotinylated JEV reporter RNA. The precipitates were analyzed by western blotting with the indicated antibodies. F) BHK‐21 cells with or without the treatment of siPABP1 were transfected with Flag‐DDX3 for 24 h and then lysed for immunoprecipitation with an anti‐Flag antibody. The protein complex was analyzed by immunoblotting. G) GST pulldown analysis of interacting partners of recombinant GST and GST‐DDX3 in BHK‐21 cell lysate. H) RNA pulldown analysis of host factors bound by nonbiotinylated or biotinylated JEV reporter RNA. The input and the precipitates were analyzed by western blotting with the indicated antibodies.

We next attempted to gain insight into the mechanisms of DDX3 and PABP1 cooperatively regulating the CI translation initiation of JEV. DDX3 and PABP1 have been reported to interact with the 40S ribosomal subunit to support the assembly of functional 80S ribosomes in eukaryotic cells.^[^ ^16^, ^17^ ^]^ The distribution of DDX3 and PABP1 in separate ribosomal subunits (40S and 60S), monosomes (80S), and larger polysomes was evaluated by polysome profiling. Based on the absorptions at 254 nm, the obtained fractions showed a distinguishable profile with peaks representing four ribosome states (**Figure** **9A**). Ribosomal protein rpS6 in the fractions containing ribosomes was detected by Western blot (Figure 9B). In cells transfected with noncapped JEV genome of ΔDB2‐sHP‐SL mutant or mock‐transfected, DDX3 mainly distributed in the top fractions without ribosomes but not in the larger polysomes fractions, while most of PABP1 sedimented in the 40S and 60S fractions and partially tailed into the 80S fractions (Figure 9B). Of note, the distribution of DDX3 shifted significantly to the fractions of 40S, 60S, and 80S ribosome states in cells transfected with noncapped JEV genome. These data supported that DDX3 specifically participates in the translation initiation phase of the noncapped JEV genome, and PABP1 is involved in the initiation phase of canonical translation.

Whether DDX3 and PABP1 function differently in CD and CI translation was further investigated. The distribution profiles of noncapped JEV mRNA and β‐actin mRNA were determined by their percentages in the fractions of BHK‐21 extracts (Figure 9C,D). The distribution of JEV mRNA and β‐actin mRNA gradually increased from monosome to polysome with peaks in fractions 10 to 12 in control cells, suggesting effective ribosome loading and continuous translation. The distribution of JEV mRNA shifted considerably toward lighter fractions with a peak at fractions 7 to 9 when DDX3 was knocked down, while the distribution of β‐actin mRNA was not affected (Figure 9C), suggesting that DDX3 is a host factor specifically required for CI translation but not for canonical translation. In contrast, the distribution of JEV mRNA and β‐actin mRNA shifted considerably toward lighter fractions when PABP1 was depleted (Figure 9D), confirming that PABP1 is critical for both CD and CI translation mechanisms.

To further clarify the function of DDX3 at the initiation step of JEV CI translation, affinity purification with the capped and noncapped biotinylated RNAs was performed to analyze translation initiation factors recruited by DDX3 to JEV 5′UTR. A range of host factors associated with translation initiation was recruited to the noncapped JEV‐UTRs, including eIF4G and eIF4A, as well as the components of 43S PIC (Figure 9E). As expected, eIF4E and p‐eIF4E were recruited to the capped RNAs but not the noncapped RNAs. In the absence of DB2 and sHP‐SL, dramatically reduced DDX3 and PABP1 were pulled down by biotinylated RNA of JEV‐UTRs, which led to the loss of other factors associated with translation initiation using the noncapped RNA. The recruitment of translation initiation complexes by DDX3 was further evaluated by co‐IP assay. DDX3 specifically coprecipitated with PABP1, eIF4G, and eIF4A, as well as eIF3A and rpS6 in the 43S complex, and PABP1 depletion alleviated these interactions (Figure 9F). The interactions between DDX3 and eIF3A/rpS6 were confirmed by the GST pull‐down assay (Figure 9G). In addition, PABP1 recruiting eIF4G to initiate translation has been demonstrated,^[^ ^51^, ^52^ ^]^ and the interaction between DDX3 and PABP1 was confirmed in Figure 6E–H.

To determine whether the formation of the DDX3/PABP1/eIF4G/eIF4A complex is dependent on JEV infection or not, we conducted additional experiments using a JEV 5′/3′UTRs with the SLA domain (DDX3‐binding region, Figure 7G) in 5′UTR deleted to analyze the complex assembly under both cap‐independent and cap‐dependent translation. We found that the ΔSLA mutant with a cap structure could form eIF4E/PABP1/eIF4G/eIF4A complex in DDX3‐WT and DDX3‐KO cell lines, thereby recruiting ribosomal 43S PIC (eIF3A/rpS6) to initiate translation (Figure 9H). Of note, noncapped ΔSLA mutant retained the ability to interact with DDX3, PABP1, and eIF4G via the 3′UTR, but failed to recruit critical translation factors (eIF4A, eIF3A) and the 40S ribosomal subunit (rpS6), resulting in the abrogation of translation initiation (Figure 9H). Therefore, the DDX3/PABP1/eIF4G/eIF4A complex required for CI translation of JEV must rely on the synergistic effect of JEV 5′UTR and 3′UTR, indicating that the formation of the DDX3/PABP1/eIF4G/eIF4A complex depends on JEV infection, rather than a spontaneous intracellular interaction.

In summary, our results suggested that DDX3 could interact with PABP1 to form DDX3/PABP1/eIF4G/eIF4A tetrameric complex, thereby recruiting 43S PIC to the 5′ end of JEV genome and allowing CI translation initiation.

## 3 Discussion

As obligate intracellular parasites, viruses exclusively rely on the host translation apparatus to support the synthesis of viral proteins. Upon infection, viruses employ diverse mechanisms to prevent the efficient expression of cellular proteins, while viral protein expression could evade these inhibitory mechanisms.^[^ ^21^, ^22^ ^]^ During flavivirus infection, the global cellular protein synthesis is almost completely repressed by PKR‐induced phosphorylation of eIF2α,^[^ ^32^, ^33^ ^]^ the inhibition of rRNA synthesis,^[^ ^53^ ^]^ or other diverse mechanisms.^[^ ^34^ ^]^ Contrary to cellular mRNA, viral mRNA is usually translated efficiently thanks to alternative translation initiation strategies. Here, we revealed that JEV translation remains efficient when the CD translation was dramatically inhibited in mammalian, avian, and mosquito cells, suggesting a possible switch of JEV translation initiation from cap‐dependent to cap‐independent under host translational shutoff. This prompted us to investigate the mechanism of the selective synthesis of viral protein in a cap‐independent manner during JEV infection.

Two alternative but not mutually exclusive cap‐independent mechanisms of translation initiation have been proposed in flaviviruses. One mechanism is IRES‐dependent initiation,^[^ ^36^, ^54^ ^]^ while the other mechanism is dependent on the 3′‐end genome.^[^ ^33^, ^35^, ^41^ ^]^ The mechanism underlying CI translation in flaviviruses remains controversial, particularly regarding its potential dependence on internal ribosome entry site (IRES)‐mediated initiation. Current debates primarily stem from comparative analyses between the CI translation efficiency of flaviviral 5′UTRs and canonical IRES elements.^[^ ^33^, ^41^ ^]^ For instance, the classification of dengue virus (DENV) and Zika virus (ZIKV) 5′UTRs as IRES elements was originally established through translational efficiency benchmarks against the hepatitis C virus (HCV) IRES.^[^ ^55^ ^]^ However, subsequent studies revealed that neither DENV nor ZIKV 5′UTRs demonstrated detectable IRES activity when inserted into the intergenic region of bicistronic reporter constructs in mosquito‐derived cell lines.^[^ ^55^ ^]^ This finding presents a paradox, given that noncapped genomic RNAs of both ZIKV and DENV maintain the capacity to produce infectious virions in mosquito cells,^[^ ^55^ ^]^ thereby suggesting the existence of an alternative, IRES‐independent translation initiation pathway in flaviviruses. Like the other flaviviruses,^[^ ^36^ ^]^ JEV can be rescued with the noncapped genomic RNA in mammalian, avian, and insect cells, confirming the CI translation of JEV. In a bicistronic reporter assay for IRES identification, the 5′UTR or 5′UTR‐cHP‐cCS of the JEV genome enabled extremely low levels of CI translation in mammalian cells. This is consistent with previously reported “IRES‐like” activities of the flavivirus 5′UTRs resulting in less than 1% of the EMCV IRES activity.^[^ ^33^, ^35^ ^]^ Thus, compared to ZIKV and DENV 5′ UTR, JEV 5′UTR does not exhibit IRES or IRES‐like activity. Despite belonging to the genus *Orthoflavivirus*, JEV, ZIKV, and DENV display low sequence similarity in their 5′UTRs,^[^ ^56^ ^]^ and this divergence may induce substantial differentiation in RNA secondary/tertiary configurations, potentially leading to variations in IRES or IRES‐like activities across these viral 5′UTRs. Of note, the typical IRES is ≈300 nucleotides in length and is characterized by complicated high‐order RNA structures assembled with stem‐loops and pseudoknots which directly recruit translation initiation factors eIF4G/4A and the 43S PIC.^[^ ^57^, ^58^ ^]^ In contrast, the length of flavivirus 5′UTR is always less than 110 nucleotides, and JEV 5′UTR can't recruit eIF4G/4A and 43S PICs. Thus, it is reasonable that the 5′UTR of JEV as well as other flaviviruses does not harbor IRES activity. Although the 5′UTR of JEV lacks IRES activity when examined alone, we cannot entirely rule out the possibility that it may form an IRES‐like structure in conjunction with downstream sequences. This requires further investigation.

Viral translation could be enhanced by the 3′UTR of their genomes.^[^ ^59^, ^60^ ^]^ In flavivirus, in addition to its role in viral RNA replication and virus production, 3′UTR serves as a translation initiation enhancer which functions similarly to the 3′‐poly(A) tail in enhancing CD translation.^[^ ^40^, ^41^, ^61^ ^]^ Intriguingly, the 3′UTR of flaviviruses can equally stimulate CI translation in the presence of flavivirus 5′UTR as well as other cellular and viral 5′UTRs. For instance, the 3′UTR of DENV stimulated the translation of reporter mRNAs containing the noncapped 5′UTRs of EMCV, BVDV, or human β‐globin,^[^ ^35^, ^40^ ^]^ and the 3′UTRs of WNV, DENV, and YFV promoted the CI translation driven by WNV 5′UTR.^[^ ^41^ ^]^ Additionally, previous studies have reported that the presence of both the DENV 5′UTR and 3′UTR is necessary to mediate resistance to the effects of CD translation suppression.^[^ ^35^ ^]^ In line with these reports, in the presence of 3′UTR, JEV 5′UTR enabled the CI translation. In addition, the CI translation mediated by JEV 5′UTR remained efficient when JEV 3′ UTR was substituted with the 3′UTRs of other flaviviruses, further supporting that the 3′UTR is essential for the CI translation of flaviviruses. In all flaviviruses, an array of RNA secondary structures has been predicted with their 3′UTRs which are ≈400 to 800 nucleotides in length, including highly complex RNA structures that are closely related to viral translation efficiency, viral replication, and pathogenicity of flaviviruses.^[^ ^8^, ^39^, ^62^ ^]^ In this study, DB2 and sHP‐SL in JEV 3′UTR were identified as key RNA structures for the CI translation of JEV. Furthermore, the recovery of rGI‐ΔDB2 with the capped viral genome but not the noncapped one, confirms the decisive role of DB2 in the CI translation of JEV. Previous studies suggested that the 3′SL of sHP‐SL enhances translation by increasing mRNAs recruited into polysomes and the number of ribosomes associated with mRNA, thereby increasing translation initiation.^[^ ^40^, ^61^ ^]^ Here, we also determined that sHL‐SL contributed to maximizing CD translation efficiency mediated by JEV 5′UTR which is indispensable for the CI translation. The deletion of DB2 resulted in decreased replication capacity of JEV in BHK‐21 cells and reduced JEV virulence in mice, while the deletion of sHP‐SL is lethal for viral viability. These results suggested that the CI translation of JEV is not only important for its evasion of host shutoff in vitro but also for its virulence in vivo.

A closed‐loop architecture is required for efficient translation of most mRNAs.^[^ ^51^, ^63^ ^]^ Similarly, the circular topology of the flaviviral RNA genome during translation has also been proposed.^[^ ^38^, ^64^ ^]^ A defining feature of flavivirus genomes is the existence of complementary sequence motifs at both termini that are involved in the genome circularization.^[^ ^42^ ^]^ JEV 5′UTR can't support CI translation without JEV 3′UTR, and JEV 3′UTR in stimulating CI translation was not affected by the absence of the cyclization sequence (CS) of 5′UTR. These observations suggested the CI translation initiation of JEV may not require direct interaction between 5′UTR and 3′ UTR.

Indeed, previous studies have proposed a translation model in which the DENV 3′UTR interacts with host proteins to deliver and/or stabilize key translation initiation factors at the 5′UTR in noncanonical translation initiation,^[^ ^35^ ^]^ yet the host translational factors engaged in non‐canonical translation initiation and their regulatory mechanisms remain incompletely elucidated.

We hypothesized that host factors recruited by JEV UTRs are required for the CI translation initiation. Therefore, a panel of cellular proteins interacting with DB2 and sHP‐SL of 3′UTR was identified. Among them, DDX3 and PABP1 were demonstrated to regulate JEV CI translation. Both DDX3 and PABP1 were considered as the general, auxiliary translation factors involved in the translation initiation process.^[^ ^16^, ^18^, ^65^ ^]^ PABP1 bound to the poly (A) tail interacts with the translation initiation complex eIF4F bound to the cap structure of mature mRNAs, thus contributing to the formation of a closed loop structure that enhances translation or promotes translation re‐initiation.^[^ ^51^ ^]^ In flavivirus infection, PABP1 could also serve as a bridging factor binding 3′UTR internally for circularization of the viral genome and translation enhancement.^[^ ^66^, ^67^ ^]^ In this study, PABP1 depletion reduced the efficiency of both CD and CI translation of JEV, supporting that PABP1 exerts a regulatory function on the translation of flaviviruses. However, PABP1 may exert distinct roles in regulating CD and CI translation of JEV. In JEV CD translation, PABP1 mainly modulates viral RNA translation efficiency. This is evidenced by the observation that deletion of the PABP1‐binding domain in the JEV 3′UTR reduced translation efficiency but preserved translation initiation. In contrast, the loss of the PABP1‐binding domain in the JEV 3′UTR completely abolished the translation initiation in CI translation, indicating that PABP1 is indispensable for JEV CI translation initiation, but not for JEV CD translation initiation. Of note, DDX3 is specifically required for the CI translation of JEV, but not for the CD translation of JEV. As reported previously,^[^ ^19^ ^]^ DDX3 binds to both UTRs of JEV mediated by the RNA structures of loop1‐stem‐loop2 in DB2 and loop1‐stem1/2‐loop2 in SLA. In line with these results, DDX3 has been proven to be required for translation driven by cellular and viral transcripts that contain complicated RNA structures in their 5′UTR but not for general translation.^[^ ^68^, ^69^ ^]^ Thus, DDX3 binds to UTRs to form a closed‐loop architecture of the JEV genome.

DDX3 is a cellular ATP‐dependent RNA helicase involved in different aspects of RNA function ranging from transcription to translation.^[^ ^70^ ^]^ An important aspect of DDX3 function is involved in translation, specifically at the initiation step.^[^ ^68^ ^]^ Of note, DDX3 appears to promote the translation of a subset of specific mRNA carrying highly structured 5′UTRs through its helicase activity which facilitates translation through the resolution of secondary structures during ribosomal scanning.^[^ ^17^, ^68^, ^71^ ^]^ However, in this study, the knockout of DDX3's helicase activity by site‐directed mutagenesis or using the specific inhibitor RK33 had no obvious effect on NS1′ expression and viral yields of noncapped JEV genome, suggesting that the RNA helicase activity of DDX3 is not important for its regulatory function in JEV CI translation. Some functions of DDX3 depend on its helicase activity, while others depend only on its direct interaction with RNA or proteins.^[^ ^72^, ^73^ ^]^ We found that the C‐terminal domain and helicase domains of DDX3 respectively mediate its interactions with PABP1 and JEV UTRs. The deletion of these domains of DDX3 led to a loss of CI translation with the noncapped JEV genome indicated by the failure of viral protein expression and virus recovery. Thus, DDX3's function in JEV CI translation is primarily dependent on its interactions with UTRs and PABP1, but not its helicase activity.

In the translation initiation phase, DDX3 could remodel the RNA structures and remove the RNA‐bound proteins of 5′UTR to facilitate 43S ribosome binding.^[^ ^17^, ^74^ ^]^ In this study, we observed higher levels of DDX3 distributed in the fractions of ribosomal subunits and monosomes in cells transfected with noncapped JEV genome, in comparison to cells without JEV CI translation. Thus, DDX3 may be actively involved in cap‐independent initiation by assisting the assembly of functional 80S ribosomes for optimal translation, which was supported by the decreased distribution of JEV mRNA in the polysome fractions of DDX3 knockdown cells. Circularization of cellular mRNAs mediated by the PABP/eIF4G binary complex brings together the cap‐binding protein eIF4E and the RNA helicase eIF4A.^[^ ^51^, ^75^ ^]^ Translation initiation is promoted by the interaction between eIF4G and eIF3 of the 43S PIC.^[^ ^51^ ^]^ The noncapped JEV‐UTRs specifically recruited PABP1, eIF4G, and eIF4A, as well as the components of the 43S PIC, which was dramatically inhibited by the deletion of DB2 and sHP‐SL in 3′UTR. In addition, DDX3 coprecipitated with eIF4G, eIF4A, PABP1, and the components of the 43S PIC. These findings support that DDX3 recruits translation initiation factors eIF4G and eIF4A to JEV UTRs via its interaction with PABP1, thereby recruiting the 43S PIC to the 5′ end of the JEV genome and allowing the initiation of CI translation.

**Figure 10:**
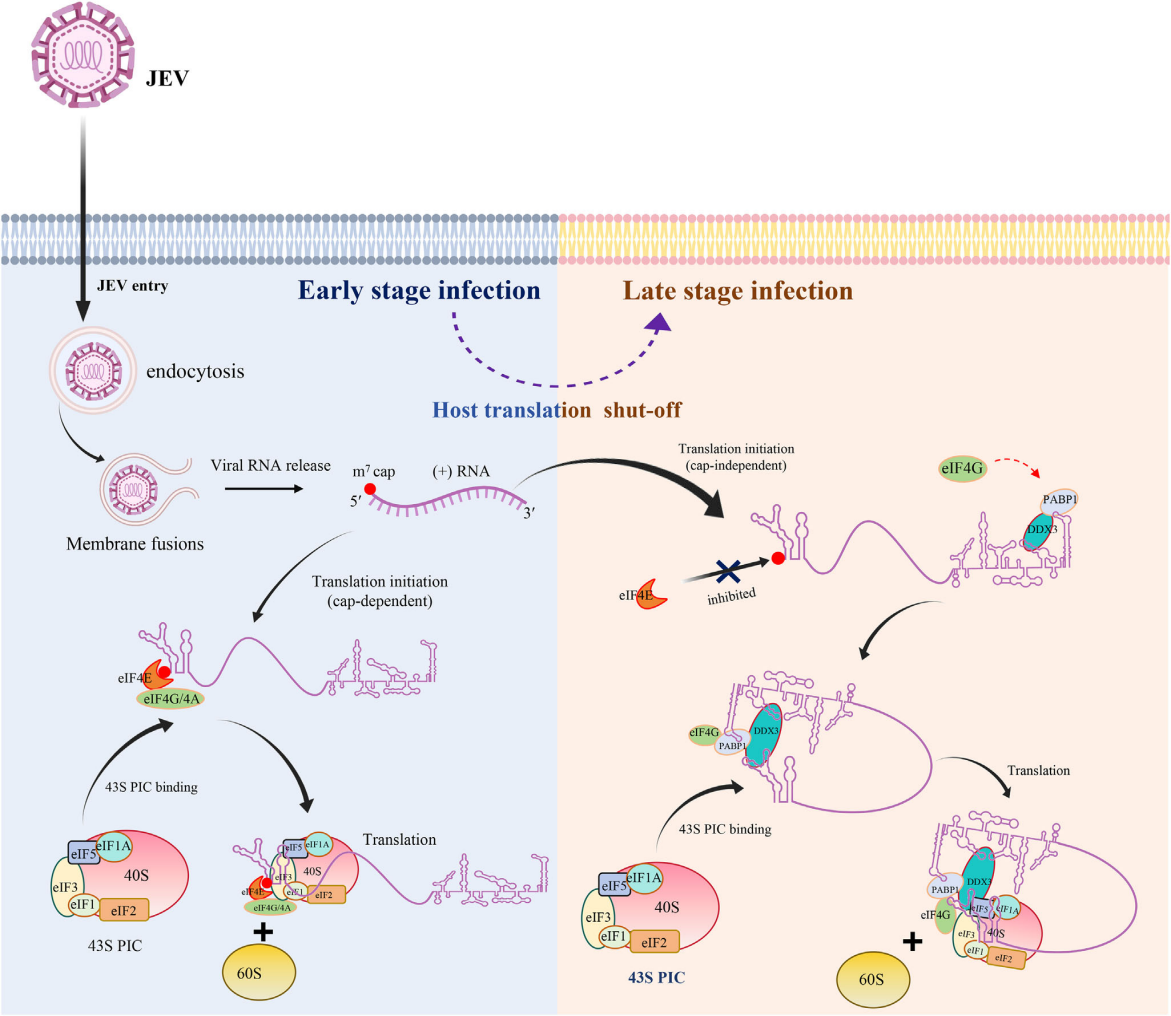
Schematic model of DDX3 and PABP1 regulation of JEV CI translation initiation. During the shut‐off of host cellular canonical translation, DDX3 and PABP1 respectively bind to *cis*‐acting elements DB2 and sHP‐SL, in which DDX3 could also anchor to 5′UTR of JEV genomic RNA to establish a closed‐loop architecture. Simultaneously, DDX3 interacts with PABP1 to form DDX3/PABP1/eIF4G/eIF4A tetrameric complex, thereby recruiting 43S PIC to the 5′end of the viral genome and allowing translation initiation.

In summary, we found that JEV employs a CI translation strategy to evade host translational shutoff. JEV 5′UTR can initiate CI translation in the presence of 3′ UTR, although it lacks IRES or IRES‐like activity. DB2 and sHP‐SL of 3′UTR were involved in the regulation of CI translation. DDX3 and PABP1 that interact with DB2 and sHP‐SL are indispensable for JEV CI translation. DDX3 binds to the loop1‐stem1‐loop2 structure of DB2 in 3′UTR and the stem‐loop1 and stem‐loop2 structures of SLA in 5′UTR, while PABP1 binds to the loop1‐loop2 structure of sHP‐SL in 3′UTR. Mechanistically, DDX3 binds to JEV UTRs to establish a closed‐loop architecture, and its interaction with PABP1 to form DDX3/PABP1/eIF4G/eIF4A tetrameric complex, thereby recruiting the 43S PIC to initiate translation (**Figure** **10**). Our findings demonstrated the CI translation mechanism of JEV which relies on the high‐order RNA structures formed by UTRs and the regulatory roles of DDX3 and PABP1. These results provide new insights into the understanding of flavivirus translation regulation.

## 4 Experimental Section

### Reagents and Antibodies

Mouse monoclonal anti‐Flag antibody was purchased from MBL. Mouse monoclonal anti‐NS1′antibody was purchased from GeneTex. Mouse monoclonal anti‐Myc and mouse anti‐puromycin antibodies were purchased from Merck Millipore. Mouse anti‐DDX3, mouse anti‐PABP1, mouse anti‐eIF4G, mouse anti‐rpS6, rabbit anti‐eIF5, and rabbit anti‐eIF1 antibodies were purchased from Proteintech Group. Rabbit anti‐β tubulin and rabbit anti‐Flag antibodies were purchased from Bioworld Technology. Mouse anti‐GST, rabbit anti‐Myc, rabbit anti‐eIF4E, rabbit anti‐Phospho‐eIF4E, rabbit anti‐Phospho‐eIF2α, rabbit anti‐eIF4A, rabbit anti‐eIF2A, rabbit anti‐eIF3A, rabbit anti‐eIF1A antibodies were purchased from ABclonal Technology. HRP‐conjugated goat anti‐mouse and HRP‐conjugated goat anti‐anti‐rabbit antibodies were purchased from BBI Life Science. Alexa Fluor 488‐AffiniPure Goat Anti‐Mouse IgG(H+L) and TRITC‐conjugated anti‐rabbit antibodies were purchased from Jackson ImmunoResearch.

The chemical reagent puromycin was purchased from Merck Millipore. The inhibitor 4E2RCat and RK‐33 were purchased from MedChemExpress.

### Cell Lines

BHK‐21, ST, DF‐1, and HEK‐293T cells were purchased from American Type Culture Collection (ATCC) and cultured in Dulbecco modified Eagle medium (DMEM, Hyclone) containing 10% fetal bovine serum (FBS, Sigma), 100 µg mL^−1^ streptomycin and 100 IU mL^−1^ penicillin (Hyclone) at 37 °C in a 5% CO_2_ incubator. C6/36 cells purchased from ATCC were grown in RPMI medium 1640 supplemented with 10% FBS, 100 µg mL^−1^ streptomycin, and 100 IU mL^−1^ penicillin at 28 °C with no additional CO_2_. For the following viral infection, all cells were maintained in a medium containing 2% FBS, 100 µg mL^−1^ streptomycin, and 100 IU mL^−1^ penicillin.

### Ethics Statement and Biosafety Procedures

All animal experiments were approved by the Institutional Animal Care and Use Committee of Yangzhou University (IACUC No: SYXK‐SU‐202202163) and were performed in compliance with the Guidelines on the Humane Treatment of Laboratory Animals (Ministry of Science and Technology of the People's Republic of China, Policy No. 2006 398). All biological experiments for JEV in this study have been approved by the Institutional Biosafety Committee (IBC) of the Yangzhou University, and performed in the biosafety level 2 facility of Yangzhou University, strictly following all biosafety management and regulation.

### Mouse Studies

Three‐week‐old C57BL/6 mice were purchased from the Laboratory Animal Center of Yangzhou University and randomly divided into four groups (10 mice per group). Mice were respectively injected intraperitoneally with rGI(P0) [rescued with ppp(5′)A‐RNA], rGI(P0) [rescued with m^7^G(5′) ppp(5′)A‐RNA] or rGI‐ΔDB2(P0) [rescued with m^7^G(5′) ppp(5′)A‐RNA] at a dose of 10^3^ or 10^5^ TCID_50_ per animal, or mock‐infected with equal volume of DMEM. All animals were monitored daily for death and clinical signs of disease.

### RNA In Vitro Transcription and Virus Recovery in Cells

The JEV infectious clones TAR‐rGI and TAR‐rGIII were constructed in the lab.^[^ ^76^ ^]^ The JEV cDNA clones for RNA in vitro transcription were generated by cleavage linearization with restriction endonuclease *Bsu36I* (NEB). The full‐length viral RNA was transcribed in vitro from the linearized JEV cDNA templates using the HiScribe T7 Quick High Yield RNA Synthesis Kit (NEB), and different 5′ cap structures of the viral genome were obtained by the addition of m^7^G(5′)ppp(5′)A cap analog or G(5′)ppp(5′)A cap analog (NEB). Two micrograms of transcript RNA were subsequently transfected into host cells seeded in a six‐well plate with Lipofectamine MessengerMAX transfection reagent (Thermo Fisher Scientific) according to the manufacturer's instructions. Culture supernatants of the transfectants exhibiting the typical cytopathic effects (CPE) of JEV infection were harvested and stored at −80 °C for further infection.

For DTMUV, a PCR‐based reverse genetics system for DTMUV was constructed (Figure 4D).^[^ ^46^ ^]^ Vial RNA was extracted from the DTMUV TA strain^[^ ^77^ ^]^ and reverse‐transcribed into cDNA. Four overlapping cDNA segments (F1: 1–2406, F2: 2366–5092, F3: 5025–8318 and F4: 8259–10991) of DTMUV were amplified by Q5 High‐Fidelity DNA Polymerase (NEB) using the specific primers (Table S4, Supporting Information). The T7 RNA polymerase promoter was introduced at the 5′ end of the F1 fragment. To generate the full‐length cDNA corresponding to the entire viral genome, a series of PCRs was performed using Q5 High‐Fidelity DNA Polymerase to prepare the four overlapping cDNA segments. The full‐length viral RNA of DTMUV was transcribed in vitro from the linearized DTMUV cDNA templates as the above method.

### Monocistronic and Bicistronic Reporters

The monocistronic reporter constructs used in this study were generated using the pGL3 vector backbone. The GI JEV 5′ UTR or GI JEV 5′UTR plus a 54‐bp‐long sequence in the 5′coding region of the C protein was respectively fused with the FLuc gene followed by either the GI JEV 3′UTR sequence or a short polyadenylated [poly(A)] tail (*n* = 7) to construct monocistronic reporters, including JEV‐5′UTR‐FLuc, JEV‐5′UTR‐FLuc‐3′UTR, JEV‐5′UTR‐cHP‐cCS‐FLuc and JEV‐5′UTR‐cHP‐cCS‐FLuc‐3′UTR. Additionally, the monocistronic reporter HCV‐5′UTR‐FLuc with the FLuc gene flanked by an HCV IRES and a poly(A) tail was used as a positive control, and the HCV‐IRES‐ΔDIII‐FLuc with the deletion of stem‐loop III was used as a negative control. As above, the reporter RNAs were transcribed using T7 polymerase to contain either an m^7^G(5′)ppp(5′)A, G(5′)ppp(5′)A or ppp(5′) 5′terminal modification.

Bicistronic reporter constructs were generated using the pRL‐SV40 vector as a backbone, including pRL‐JEV‐5′UTR‐FLuc, pRL‐JEV‐5′UTR‐cHP‐cCS‐FLuc, pRL‐HCV‐IRES‐FLuc, and pRL‐HCV‐IRES‐ΔDIII‐FLuc. The upstream RLuc was flanked by an SV40 promoter and two stop codons, while the downstream FLuc was flanked by GI JEV 5′ UTR, JEV 5′UTR plus a 54‐bp N‐terminal coding region of the C protein, an HCV IRES or an HCV IRES‐ΔDIII and a stop codon.

### Luciferase Reporter Assay

For the bicistronic reporter assay, the bicistronic pRL‐JEV‐5′UTR, pRL‐JEV‐5′UTR‐cHP‐cCS‐FLuc, pRL‐HCV‐IRES or pRL‐HCV‐IRES‐ΔDIII reporter plasmids were respectively transfected into HEK‐293T cells for 24 h. The cells were collected and lysed in 1 × cell lysis buffer, after which the activities of RLuc and FLuc in cell lysates were detected in a luminometer (SuperMax 3000FL) using a dual‐luciferase reporter assay system (Vazyme). For the monocistronic expression assay, 1 µg of RNA transcripts of JEV‐5′UTR‐FLuc, JEV‐5′ UTR‐FLuc‐3′UTR, JEV‐5′UTR‐cHP‐cCS‐FLuc or JEV‐5′UTR‐cHP‐cCS‐FLuc‐3′UTR was respectively co‐transfected into BHK‐21 cells with a 5′ capped‐RLuc mRNA using Lipofectamine MessengerMax reagent (Thermo Fisher Scientific). At 12 hpt, the luciferase activity of FLuc was measured by the addition of FLuc substrate (Vazyme).

The *gaussia* luciferase assay was performed as previously described.^[^ ^78^ ^]^ Briefly, culture supernatants of cells transfected with JEV replicon RNA were harvested and cleared by centrifugation at 4 °C. The luciferase activity was determined in a luminometer (LumiStation‐1800) by mixing 20 µL culture supernatant with 50 µL reaction substrate Coelenterazine h (20 µm, pH 7.2; Maokang Biotechnology).

### Immunoblotting and Indirect Immunofluorescence Assay

Immunoblotting analysis was performed as previously described.^[^ ^20^ ^]^ Briefly, whole cell extracts were lysed in ice‐cold RIPA lysis buffer (Biosharp) with protease inhibitor (Roche). The supernatants of cell lysates were collected by centrifugation, separated by 10% SDS‐PAGE, and further transferred onto a nitrocellulose (NC) membrane (Cytiva). The membrane was blocked with 5% skim milk in PBS and then respectively incubated with primary and secondary antibodies. The visualization of protein bands was performed using the Tanon 5200 Multi chemiluminescent imaging system (Tanon). For indirect immunofluorescence assay, cells were fixed in 4% paraformaldehyde for 1 h, permeabilized with 0.05% NP‐40 for 15 min, and blocked with 5% bovine serum albumin (BSA) for 1 h at 37 °C. Subsequently, cells were incubated with primary antibody for 1 h, followed by secondary antibodies for 1 h. The nuclei were stained with 4,6‐diamidino‐2‐phenylindole (DAPI) (Solarbio) for 15 min and the fluorescence images of cells were taken with a fluorescence microscope (Carl Zeiss).

### CRISPR‐Cas9‐Based Genome Editing

DDX3‐KO cells were generated using the CRISPR/Cas9 editing method. A guide RNA targeting DDX3 DNA (5′‐AGTGGAAAATGCGCTCGGGC‐3′) was designed using E‐CRISP (http://www.e‐crisp.org/E‐CRISP/designcrispr.html). The gene knockout cells with green fluorescence were sorted into 96‐well plates by FACS and confirmed by immunoblotting and sequencing the genomic DNA.

### RNA Pulldown Assay

RNA pulldown assay was performed as described in the previous study.^[^ ^20^ ^]^ In brief, BHK‐21 or 293T cells were lysed with cell lysis buffer containing 200 U mL^−1^ RNasin (Beyotime), and centrifugation at 16 000 g for 10 min at 4 °C was performed to remove cell debris. The biotinylated RNA was synthesized using the T7 polymerase with the addition of 1.25 µL of 20 mm Biotin‐16‐UTP (Roche). The biotinylated RNA was heated to 90 °C for 2 min in RNA folding buffer (10 mm Tris [pH7], 0.1 m KCl, and 10 mm MgCl_2_) and the mixture was shifted to room temperature for 20 min to allow proper secondary structure formation. For the biotinylated RNA‐binding assay, a reaction mixture containing 500 µg of cell extract and 2 µg of biotinylated RNA was prepared. The mixture, at a final volume of 100 µL, was incubated in the reaction solution (5 mm HEPES [pH7.1], 40 mm KCl, 2 mm MgCl_2,_ and 1U RNasin) for 2 h at 30 °C and then incubated with 100 µL of streptavidin magnetic beads (NEB) for 30 min at room temperature. The RNA‐protein complexes were washed five times with reaction solution buffer. The RNA binding proteins were eluted by boiling the beads in 0.1% SDS and were analyzed by immunoblotting or mass spectrometry.

### GST Pull‐Down Assay

GST‐DDX3, GST‐PABP1, and GST proteins expressed in *E. coli* were purified with GSH Magnetic Agarose Beads (Beyotime). Ten micrograms of purified GST‐DDX3, GST‐PABP1, or GST protein was incubated with HEK‐293T cell lysates with the expression of PABP1‐Myc or DDX3‐Flag for 8 h at 4 °C. The protein complexes were precipitated with GSH Magnetic Agarose Beads and detected with anti‐GST, anti‐Myc, and anti‐Flag antibodies.

### RNA Immunoprecipitation (RIP) Followed by RT‐qPCR (RIP‐qPCR)

A total of 2 × 10^7^ BHK‐21 cells were infected with rGI or rGI‐ΔDB2 at an MOI of 0.01 or mock‐infected. At 12, 24, and 36 hpi, the culture medium was replaced with cold 1 × PBS and cell monolayers were irradiated with short‐wave UV light at 5700 × 100 µJ per cm^2^. After cross‐linking, the cells were harvested by scraping and centrifugation before being lysed in RIPA buffer (50 mm Tris [pH 7.6], 150 mm NaCl, 1% NP‐40, 0.5% sodium deoxycholate, 0.1% SDS). The cell lysate was then incubated for 4 h with either mouse anti‐DDX3 antibody or mouse IgG‐linked beads at 4 °C. Subsequently, RNA captured by RIP was recovered using the Total RNA Extraction Reagent (Vazyme). The enrichment of JEV mRNA in each sample was assessed via RT‐qPCR using specific primers (Table S4, Supporting Information). The outcomes were calculated by normalization of RNA abundance in RIP samples (IgG and DDX3 mutant) to respective inputs.

### Polysome Profile Analysis

Cells were incubated with 0.1 mg mL^−1^ CHX (Sigma) for 5 min at 37 °C and then lysed in polysomal extraction buffer (20 mm Tris‐HCl [pH 7.5], 5 mm MgCl2, 100 mm KCl, 1% Triton X‐100, 0.1 mg mL^−1^ CHX, 1 × protease inhibitor cocktail, and 50 U mL^−1^ RNase inhibitor) for polysome fractionation by sucrose density gradient ultracentrifugation according to previous reports.^[^ ^20^ ^]^ After centrifugation, each 500 µL fraction was collected and analyzed by UV absorbance at 254 nm. For analysis of proteins in polysomes, total proteins from each sucrose gradient fraction were precipitated with trichloroacetic acid (TCA) and analyzed by immunoblotting. To examine the distribution of mRNA in the gradients, total RNA was extracted with TRIzol (Vazyme) for quantitative RT‐PCR analysis using specific primers targeting JEV and β‐actin cDNAs (Table S4, Supporting Information).

### Secondary Structure Modeling

The secondary structure of JEV UTR was predicted with the Mfold software v 3.6 (https://www.unafold.org/mfold/applications/rna‐folding‐form‐php) under the parameters of folding temperature at 37 °C, an ionic condition of 1 m NaCl with no divalent ions, and the VARNA program (https://varna.lri.fr/) was employed to modify the RNA structures.

### RNA Helicase Activity Assay

A fluorescence resonance energy transfer^[^ ^79^ ^]^ was used to detect DDX3 helicase activity (Figure S10, Supporting Information). The following fluorescently labeled single‐stranded RNA fragments based on the sequence of loop1‐stem‐loop2 in DB2 of JEV 3′UTR (Figures S9A,S10, Supporting Information) were chemically synthesized by GenScript Biotech (Nanjing): 5′(BHQ‐2‐dT)‐CCAGCAAACCCUCGAAGCUGAUGAGGGUGGAGCUGG‐(Cy5)‐3′, and then slowly annealed by PCR (95 °C, 20 min; 60 °C, 10 min; 37 °C, 10 min; 20 °C, 10 min) to form a dsRNA substrate. The capture DNA chain 5′‐GAGGGTTTGCTGC‐3′ was synthesized and used to immediately capture the 5′ sequences of labeled single‐stranded RNA fragments to prevent the formation of double‐stranded RNA. The dsRNA substrate (4 µmol L^−1^) and capture DNA chain (100 µmol L^−1^) were co‐incubated with purified GST‐DDX3 or GST‐DDX3‐Mut (50 µmol L^−1^), MgCl_2_ (50 mmol L^−1^), and ATP (10 mmol L^−1^). Initial fluorescence in the reaction system in a 96‐well plate was detected with a multifunction microplate reader at an excitation wavelength of 625 nm (excitation) and an emission wavelength of 670 nm (emission). The unwinding reaction was carried out at 37 °C. Four replicates from each experimental group were assessed at different time points.

### Statistical Analysis

Statistical analysis was conducted using the unpaired Student's *t*‐test, or one‐way analysis of variance (ANOVA) with Bonferroni's post‐tests for multiple‐group comparisons. Animal survival analysis employed the Kaplan–Meier method to generate graphs and log‐rank analysis was used to analyze the survival curves. *P‐*values less than 0.05 were considered statistically significant.

## Conflict of Interest

The authors declare no conflict of interest.

## Author Contributions

C.L., Y.L., and M.S. designed experiments and interpreted the data. L.Z., C.T., X.C., J.S., Q.L., X.J., J.G., and B.W. performed experiments and analyzed the data. K.B., A.W., and Y.Y. discussed the data. C.L. and Y.L. conceived the study, supervised the work, and wrote the paper. All authors read and approved the final manuscript.

## Supporting information



Supporting Information

## Data Availability

The data that support the findings of this study are available from the corresponding author upon reasonable request;

## References

[1] S. W. van Leur , T. Heunis , D. Munnur , S. Sanyal , Virulence 2021, 12, 2814. 34696709 10.1080/21505594.2021.1996059 PMC8632085

[2] T. E. Erlanger , S. Weiss , J. Keiser , J. Utzinger , K. Wiedenmayer , Emerg. Infect. Dis. 2009, 15, 1. 19116041 10.3201/eid1501.080311 PMC2660690

[3] Y. C. Fan , J. J. Liang , J. M. Chen , J. W. Lin , Y. Y. Chen , K. H. Su , C. C. Lin , W. C. Tu , M. T. Chiou , S. C. Ou , G. J. Chang , Y. L. Lin , S. S. Chiou , PLoS Pathog. 2019, 15, 1007992. 10.1371/journal.ppat.1007992 PMC6695206 31381617

[4] C. Xiao , C. Li , D. Di , J. Cappelle , L. Liu , X. Wang , L. Pang , J. Xu , K. Liu , B. Li , D. Shao , Y. Qiu , W. Ren , F. Widén , V. Chevalier , J. Wei , X. Wu , Z. Ma , PLoS Negl. Trop. Dis. 2018, 12, 0007046. 10.1371/journal.pntd.0007046 PMC6314627 30562354

[5] M. Yamada , K. Nakamura , M. Yoshii , Y. Kaku , M. Narita , J. Comp. Pathol. 2009, 141, 156. 19523649 10.1016/j.jcpa.2009.04.006

[6] H. Ladreyt , B. Durand , P. Dussart , V. Chevalier , Viruses 2019, 11, 949. 31618959 10.3390/v11100949 PMC6832429

[7] D. Di , C. Li , J. Zhang , M. Hameed , X. Wang , Q. Xia , H. Li , S. Xi , Z. Li , K. Liu , B. Li , D. Shao , Y. Qiu , J. Wei , Z. Ma , Pathogens 2020, 9, 371. 32408553 10.3390/pathogens9050371 PMC7281460

[8] Q. Y. Zhang , S. Q. Liu , X. D. Li , J. Q. Li , Y. N. Zhang , C. L. Deng , H. L. Zhang , X. F. Li , C. X. Fang , F. X. Yang , B. Zhang , Y. Xu , H. Q. Ye , Emerg. Microbes Infect. 2022, 11, 123. 34877923 10.1080/22221751.2021.2016354 PMC8725919

[9] X. Qiu , Y. Lei , P. Yang , Q. Gao , N. Wang , L. Cao , S. Yuan , X. Huang , Y. Deng , W. Ma , T. Ding , F. Zhang , X. Wu , J. Hu , S. L. Liu , C. Qin , X. Wang , Z. Xu , Z. Rao , Nat. Microbiol. 2018, 3, 287. 29379207 10.1038/s41564-017-0099-x

[10] T. J. Chambers , C. S. Hahn , R. Galler , C. M. Rice , Annu. Rev. Microbiol. 1990, 44, 649. 2174669 10.1146/annurev.mi.44.100190.003245

[11] C. Li , D. Di , H. Huang , X. Wang , Q. Xia , X. Ma , K. Liu , B. Li , D. Shao , Y. Qiu , Z. Li , J. Wei , Z. Ma , PLoS Pathog. 2020, 16, 1008773. 10.1371/journal.ppat.1008773 PMC7494076 32881988

[12] N. Han , J. Adams , P. Chen , Z. Y. Guo , X. F. Zhong , W. Fang , N. Li , L. Wen , X. Y. Tao , Z. M. Yuan , S. Rayner , J. Virol. 2014, 88, 11469. 25056890 10.1128/JVI.02050-14 PMC4178791

[13] V. Gandin , B. P. English , M. Freeman , L. P. Leroux , S. Preibisch , D. Walpita , M. Jaramillo , R. H. Singer , Nat. Commun. 2022, 13, 6558. 36323665 10.1038/s41467-022-34052-8 PMC9630388

[14] N. Sonenberg , A. G. Hinnebusch , Cell 2009, 136, 731. 19239892 10.1016/j.cell.2009.01.042 PMC3610329

[15] R. J. Jackson , C. U. Hellen , T. V. Pestova , Nat. Rev. Mol. Cell Biol. 2010, 11, 113. 20094052 10.1038/nrm2838 PMC4461372

[16] R. Geissler , R. P. Golbik , S. E. Behrens , Nucleic Acids Res. 2012, 40, 4998. 22323517 10.1093/nar/gks070 PMC3367175

[17] R. Soto‐Rifo , P. S. Rubilar , T. Limousin , S. de Breyne , D. Décimo , T. Ohlmann , EMBO J. 2012, 31, 3745. 22872150 10.1038/emboj.2012.220 PMC3442272

[18] C. S. Lee , A. P. Dias , M. Jedrychowski , A. H. Patel , J. L. Hsu , R. Reed , Nucleic Acids Res. 2008, 36, 4708. 18628297 10.1093/nar/gkn454 PMC2504307

[19] C. Li , L. L. Ge , P. P. Li , Y. Wang , J. J. Dai , M. X. Sun , L. Huang , Z. Q. Shen , X. C. Hu , H. Ishag , X. Mao , Virology 2014, 449, 70. 24418539 10.1016/j.virol.2013.11.008 PMC7111930

[20] S. Han , S. Sun , P. Li , Q. Liu , Z. Zhang , H. Dong , M. Sun , W. Wu , X. Wang , H. Guo , J. Virol. 2020, 94, 01679. 10.1128/JVI.01679-19 PMC6955262 31619563

[21] D. Walsh , M. B. Mathews , I. Mohr , Cold Spring Harbor Perspect. Biol. 2013, 5, 012351. 10.1101/cshperspect.a012351 PMC3579402 23209131

[22] D. Walsh , I. Mohr , Nat. Rev. Microbiol. 2011, 9, 860. 22002165 10.1038/nrmicro2655 PMC7097311

[23] E. Royall , N. Doyle , A. Abdul‐Wahab , E. Emmott , S. J. Morley , I. Goodfellow , L. O. Roberts , N. Locker , J. Biol. Chem. 2015, 290, 4748. 25561727 10.1074/jbc.M114.602649 PMC4335213

[24] T. P. Herbert , I. Brierley , T. D. Brown , J. Gen. Virol. 1997, 78, 1033. 9152420 10.1099/0022-1317-78-5-1033

[25] T. Yokoyama , K. Machida , W. Iwasaki , T. Shigeta , M. Nishimoto , M. Takahashi , A. Sakamoto , M. Yonemochi , Y. Harada , H. Shigematsu , M. Shirouzu , H. Tadakuma , H. Imataka , T. Ito , Mol. Cell 2019, 74, 1205. 31080011 10.1016/j.molcel.2019.04.022

[26] J. M. Burks , C. Zwieb , F. Müller , I. K. Wower , J. Wower , Virus Res. 2011, 160, 136. 21683744 10.1016/j.virusres.2011.06.002

[27] N. Locker , L. E. Easton , P. J. Lukavsky , EMBO J. 2007, 26, 795. 17255934 10.1038/sj.emboj.7601549 PMC1794401

[28] T. D. Plank , J. T. Whitehurst , J. S. Kieft , Nucleic Acids Res. 2013, 41, 6698. 23661682 10.1093/nar/gkt358 PMC3711417

[29] U. Bhardwaj , P. Powell , D. J. Goss , Nucleic Acids Res. 2019, 47, 6225. 31114905 10.1093/nar/gkz448 PMC6614841

[30] M. Chattopadhyay , M. M. Kuhlmann , K. Kumar , A. E. Simon , Virology 2014, 458‐459, 43. 10.1016/j.virol.2014.03.027 PMC4101382 24928038

[31] M. R. Fabian , K. A. White , J. Biol. Chem. 2004, 279, 28862. 15123633 10.1074/jbc.M401272200

[32] Y. C. Tu , C. Y. Yu , J. J. Liang , E. Lin , C. L. Liao , Y. L. Lin , J. Virol. 2012, 86, 10347. 22787234 10.1128/JVI.00525-12 PMC3457255

[33] T. Wang , A. Merits , Y. Wu , M. Wang , R. Jia , D. Zhu , M. Liu , X. Zhao , Q. Yang , Y. Wu , S. Zhang , Y. Liu , L. Zhang , Y. Yu , L. Pan , S. Chen , A. Cheng , J. Virol. 2020, 94, 00906. 10.1128/JVI.00906-20 PMC7394898 32522848

[34] H. Roth , V. Magg , F. Uch , P. Mutz , P. Klein , K. Haneke , V. Lohmann , R. Bartenschlager , O. T. Fackler , N. Locker , G. Stoecklin , A. Ruggieri , mBio 2017, 8, 00488. 10.1128/mBio.00488-17 PMC5395670 28420740

[35] D. Edgil , C. Polacek , E. Harris , J. Virol. 2006, 80, 2976. 16501107 10.1128/JVI.80.6.2976-2986.2006 PMC1395423

[36] Y. Song , J. Mugavero , C. B. Stauft , E. Wimmer , mBio 2019, 10, 00459. 10.1128/mBio.00459-19 PMC6456755 30967466

[37] N. J. Barrows , R. K. Campos , K. C. Liao , K. R. Prasanth , R. Soto‐Acosta , S. C. Yeh , G. Schott‐Lerner , J. Pompon , O. M. Sessions , S. S. Bradrick , M. A. Garcia‐Blanco , Chem. Rev. 2018, 118, 4448. 29652486 10.1021/acs.chemrev.7b00719 PMC5937540

[38] W. W. Chiu , R. M. Kinney , T. W. Dreher , J. Virol. 2005, 79, 8303. 15956576 10.1128/JVI.79.13.8303-8315.2005 PMC1143759

[39] L. Upstone , R. Colley , M. Harris , N. Goonawardane , PLoS Negl. Trop. Dis. 2023, 17, 0011098. 10.1371/journal.pntd.0011098 PMC9894543 36689554

[40] K. L. Holden , E. Harris , Virology 2004, 329, 119. 15476880 10.1016/j.virol.2004.08.004

[41] A. Berzal‐Herranz , B. Berzal‐Herranz , S. E. Ramos‐Lorente , C. Romero‐López , Int. J. Mol. Sci. 2022, 23, 8604. 35955738 10.3390/ijms23158604 PMC9369090

[42] D. E. Alvarez , A. L. De Lella Ezcurra , S. Fucito , A. V. Gamarnik , Virology 2005, 339, 200. 16002117 10.1016/j.virol.2005.06.009

[43] C. Li , L. Zhang , X. Chen , D. Jiang , J. Hu , J. Guo , J. Ding , X. Jiao , W. Bao , Y. Li , Antiviral Res. 2023, 216, 105652. 37301446 10.1016/j.antiviral.2023.105652

[44] S. I. Yun , Y. J. Choi , B. H. Song , Y. M. Lee , J. Virol. 2009, 83, 7909. 19494005 10.1128/JVI.02541-08 PMC2715749

[45] L. Yu , L. Markoff , J. Virol. 2005, 79, 2309. 15681432 10.1128/JVI.79.4.2309-2324.2005 PMC546603

[46] X. Wu , Y. Shi , D. Yan , X. Li , P. Yan , X. Gao , Y. Zhang , L. Yu , C. Ren , G. Li , L. Yan , Q. Teng , Z. Li , PLoS One 2016, 11, 0156579. 10.1371/journal.pone.0156579 PMC4889061 27248497

[47] P. T. Winnard , F. Vesuna , V. Raman , Antiviral Res. 2021, 185, 104994. 33301755 10.1016/j.antiviral.2020.104994 PMC9667528

[48] H. Wang , S. Kim , W. S. Ryu , J. Virol. 2009, 83, 5815. 19297497 10.1128/JVI.00011-09 PMC2681949

[49] V. S. Yedavalli , C. Neuveut , Y. H. Chi , L. Kleiman , K. T. Jeang , Cell 2004, 119, 381. 15507209 10.1016/j.cell.2004.09.029

[50] M. Xie , F. Vesuna , S. Tantravedi , G. M. Bol , M. R. Heerma van Voss , K. Nugent , R. Malek , K. Gabrielson , P. J. van Diest , P. T. Tran , V. Raman , Cancer Res. 2016, 76, 6340. 27634756 10.1158/0008-5472.CAN-16-0440 PMC5576499

[51] A. Kahvejian , G. Roy , N. Sonenberg , Cold Spring Harbor Symp. Quant. Biol. 2001, 66, 293. 12762031 10.1101/sqb.2001.66.293

[52] S. Ma , R. B. Bhattacharjee , J. Bag , FEBS J. 2009, 276, 552. 19087191 10.1111/j.1742-4658.2008.06803.x

[53] M. Selinger , H. Tykalová , J. Štěrba , P. Věchtová , Z. Vavrušková , J. Lieskovská , A. Kohl , E. Schnettler , L. Grubhoffer , PLoS Negl. Trop. Dis. 2019, 13, 0007745. 10.1371/journal.pntd.0007745 PMC6785130 31560682

[54] L. Fernández‐García , J. Angulo , H. Ramos , A. Barrera , K. Pino , J. Vera‐Otarola , M. López‐Lastra , J. Virol. 2021, 95, 01998. 10.1128/JVI.01998-20 PMC8092825 33298544

[55] L. A. Byk , N. G. Iglesias , F. A. De Maio , L. G. Gebhard , M. Rossi , A. V. Gamarnik , mBio 2016, 7, 00804. 10.1128/mBio.00804-16 PMC4937216 27353759

[56] A. A. Khromykh , H. Meka , K. J. Guyatt , E. G. Westaway , J. Virol. 2001, 75, 6719. 11413342 10.1128/JVI.75.14.6719-6728.2001 PMC114398

[57] K. M. Lee , C. J. Chen , S. R. Shih , Trends Microbiol. 2017, 25, 546. 28242053 10.1016/j.tim.2017.01.010

[58] M. E. Filbin , J. S. Kieft , Curr. Opin. Struct. Biol. 2009, 19, 267. 19362464 10.1016/j.sbi.2009.03.005 PMC2757110

[59] M. Rasekhian , F. Roohvand , S. Habtemariam , M. Marzbany , M. Kazemimanesh , Mini‐Rev. Med. Chem. 2021, 21, 2389. 33596798 10.2174/1389557521666210217092305

[60] B. Mazumder , V. Seshadri , P. L. Fox , Trends Biochem. Sci. 2003, 28, 91. 12575997 10.1016/S0968-0004(03)00002-1

[61] S. E. Ramos‐Lorente , B. Berzal‐Herranz , C. Romero‐López , A. Berzal‐Herranz , Virus Res. 2024, 343, 199340. 38387694 10.1016/j.virusres.2024.199340 PMC10907855

[62] S. M. Villordo , J. M. Carballeda , C. V. Filomatori , A. V. Gamarnik , Trends Microbiol. 2016, 24, 270. 26850219 10.1016/j.tim.2016.01.002 PMC4808370

[63] M. K. Thompson , W. V. Gilbert , Curr. Genet. 2017, 63, 613. 28028558 10.1007/s00294-016-0674-3 PMC5484751

[64] R. M. Kofler , V. M. Hoenninger , C. Thurner , C. W. Mandl , J. Virol. 2006, 80, 4099. 16571826 10.1128/JVI.80.8.4099-4113.2006 PMC1440478

[65] J. Wang , X. Zhang , G. H. Greene , G. Xu , X. Dong , Cell 2022, 185, 3186. 35907403 10.1016/j.cell.2022.06.037 PMC9391319

[66] C. Polacek , P. Friebe , E. Harris , J. Gen. Virol. 2009, 90, 687. 19218215 10.1099/vir.0.007021-0

[67] T. J. Shen , C. L. Chen , T. T. Tsai , M. K. Jhan , C. H. Bai , Y. C. Yen , C. W. Tsai , C. Y. Lee , P. C. Tseng , C. Y. Yu , C. F. Lin , JCI Insight 2022, 7, 142805. 10.1172/jci.insight.142805 PMC9675471 36125898

[68] M. C. Lai , Y. H. Lee , W. Y. Tarn , Mol. Biol. Cell 2008, 19, 3847. 18596238 10.1091/mbc.E07-12-1264 PMC2526709

[69] L. E. Ayalew , A. K. Patel , A. Gaba , A. Islam , S. K. Tikoo , Front. Microbiol. 2016, 7, 2119. 28082972 10.3389/fmicb.2016.02119 PMC5186766

[70] J. Mo , H. Liang , C. Su , P. Li , J. Chen , B. Zhang , Mol Cancer 2021, 20, 38. 33627125 10.1186/s12943-021-01325-7 PMC7903766

[71] M. C. Lai , W. C. Chang , S. Y. Shieh , W. Y. Tarn , Mol. Cell. Biol. 2010, 30, 5444. 20837705 10.1128/MCB.00560-10 PMC2976371

[72] A. A. Putnam , E. Jankowsky , Biochim. Biophys. Acta 2013, 1829, 884. 23416748 10.1016/j.bbagrm.2013.02.002 PMC3661757

[73] T. Hernández‐Díaz , F. Valiente‐Echeverría , R. Soto‐Rifo , Microorganisms 2021, 9, 1206. 34204859 10.3390/microorganisms9061206 PMC8227550

[74] L. Calviello , S. Venkataramanan , K. J. Rogowski , E. Wyler , K. Wilkins , M. Tejura , B. Thai , J. Krol , W. Filipowicz , M. Landthaler , S. N. Floor , Nucleic Acids Res. 2021, 49, 5336. 33905506 10.1093/nar/gkab287 PMC8136831

[75] S. E. Wells , P. E. Hillner , R. D. Vale , A. B. Sachs , Mol. Cell 1998, 2, 135. 9702200 10.1016/s1097-2765(00)80122-7

[76] C. Li , X. Chen , Y. Zhou , J. Hu , X. Wang , Y. Li , Antiviral Res. 2022, 197, 105233. 34973281 10.1016/j.antiviral.2021.105233

[77] X. Chen , C. Li , W. Lin , T. Li , X. Li , X. Bai , S. Wulin , Q. Zhang , S. Li , M. Liu , J. H. Liu , Y. Zhang , J. Immunol. 2020, 204, 1836. 32132180 10.4049/jimmunol.1901352 PMC7086385

[78] C. Li , X. Chen , J. Hu , D. Jiang , D. Cai , Y. Li , Int. J. Mol. Sci. 2022, 23, 15548. 36555192 10.3390/ijms232415548 PMC9778660

[79] Y. Shi , X. Tong , G. Ye , R. Xiu , L. Li , L. Sun , J. Shi , M. Li , Y. Song , C. Fan , K. Shi , Z. F. Fu , S. Xiao , G. Peng , J. Virol. 2020, 94, 02158. 10.1128/JVI.02158-19 PMC7375384 32461315

