# Supplementary material for: DDX3 Regulates the Cap‐Independent Translation of the Japanese Encephalitis Virus via Its Interactions with PABP1 and the Untranslated Regions of the Viral Genome
Source: Adv Sci (Weinh). 2025 May 8;12(27):2502493. doi: 10.1002/advs.202502493 (PMC12279179; doi:10.1002/advs.202502493)
Supplement: Supplementary file 1 — Supporting Information [file ADVS-12-2502493-s001.docx]

Supporting Information

**DDX3 regulates the cap-independent translation of the Japanese encephalitis virus via its interactions with PABP1 and the untranslated regions of the viral genome**

*Chenxi Li, Linjie Zhang, Chenyang Tang, Xuan Chen, Jing Shi, Qingyu Li, Xue Jiao, Jinyao Guo, Bin Wang, Kefan Bu,* *Abdul Wahaab, Yuguo Yuan, Ming-an Sun, Yanhua Li* ^*^


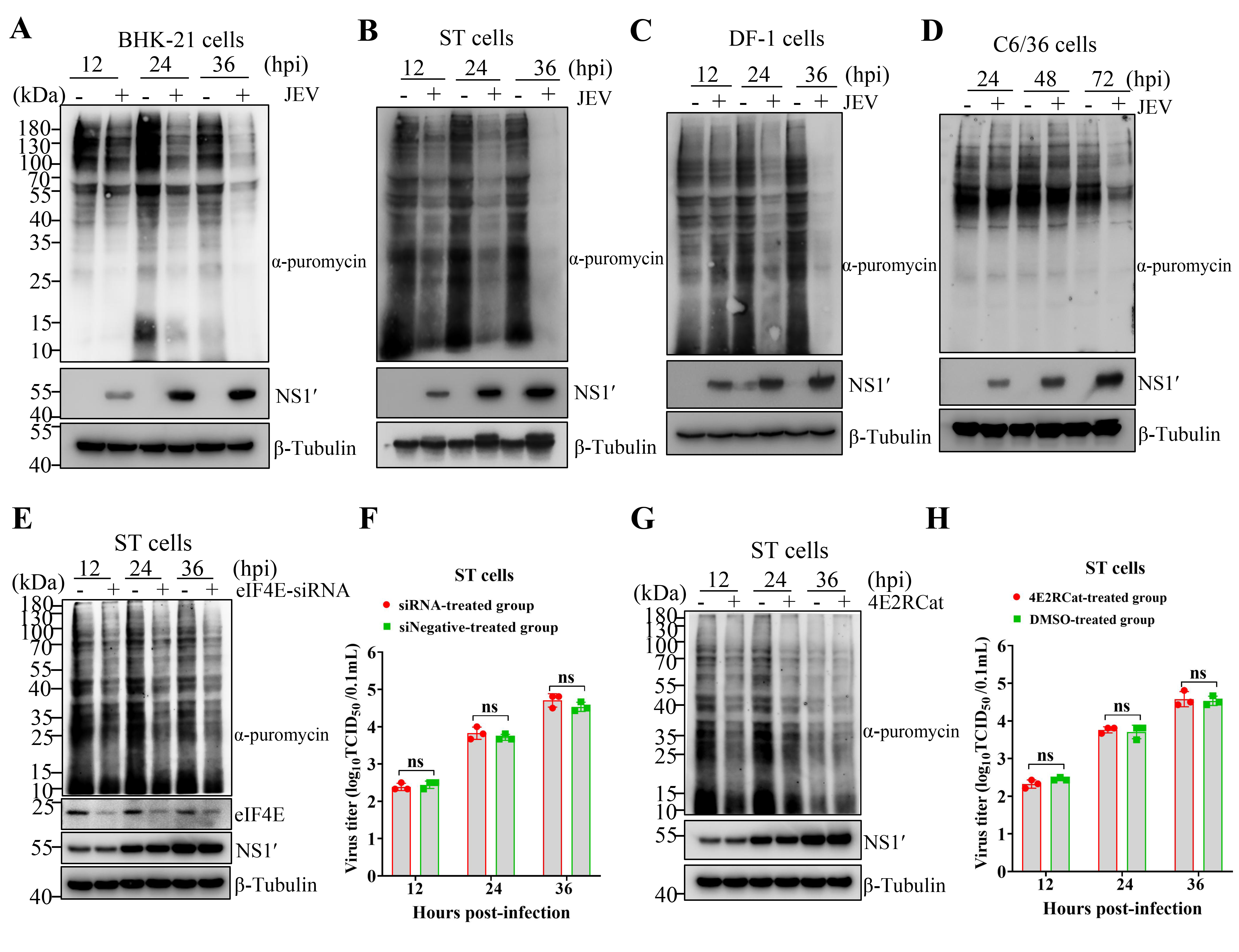


**Figure S1.** JEV infection was not restricted by the suppression of cap-dependent translation initiation. A to D) The puromycin incorporation assay of BHK-21, ST and DF-1 cells infected with 0.05 MOI JEV for 12, 24 and 36 h, or C6/36 cells infected with 0.05 MOI JEV for 24, 48 and 72 h. E,G) The puromycin incorporation assay at 12, 24 and 36 hpi of JEV in ST cells treated with 100 pmol eIF4E-specific siRNA (E) or 20 μM 4E2RCat (G). F,H) The viral titers in the supernatants of BHK-21 cells treated with eIF4E-specific siRNAs (F) or 4E2RCat (H). ns, no statistical differences; Data are presented as mean ± standard deviation (SD) and statistically significant tested by Student’s *t*-test (F and H).

**
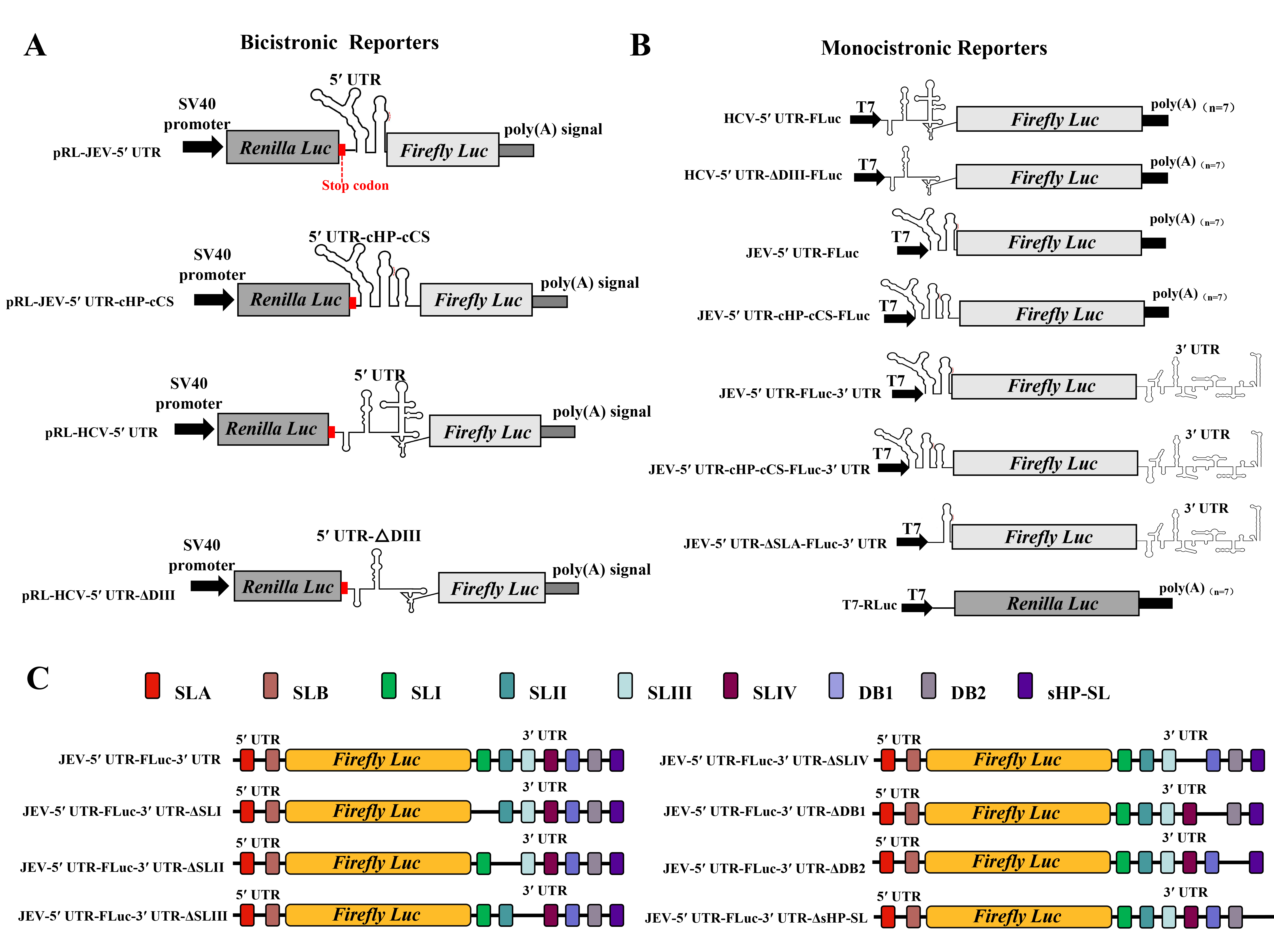
**

**Figure S2.** Schematic diagram of reporters constructed for the identification of key elements for JEV cap-independent initiation. A) Schematic illustration of bicistronic constructs pRL-JEV-5′UTR, pRL-JEV-5′UTR-cHP-cCS, pRL-HCV-5′UTR and pRL-HCV-5′UTR-Δdomain III. B) Diagrams of JEV monocistronic reporter constructs controlled by T7 promoter: HCV-5′UTR-FLuc, HCV-5′UTR-Δdomain III-FLuc, JEV-5′UTR-FLuc, JEV-5′UTR-cHP-cCS-FLuc, JEV-5′UTR-FLuc-3′UTR, JEV-5′UTR-cHP-cCS-FLuc-3′UTR and JEV-5′UTR-ΔSLA-FLuc-3′UTR. C) Diagrams of JEV monocistronic reporter constructs with the deletion of each *cis*-acting element within 3′UTR.

**
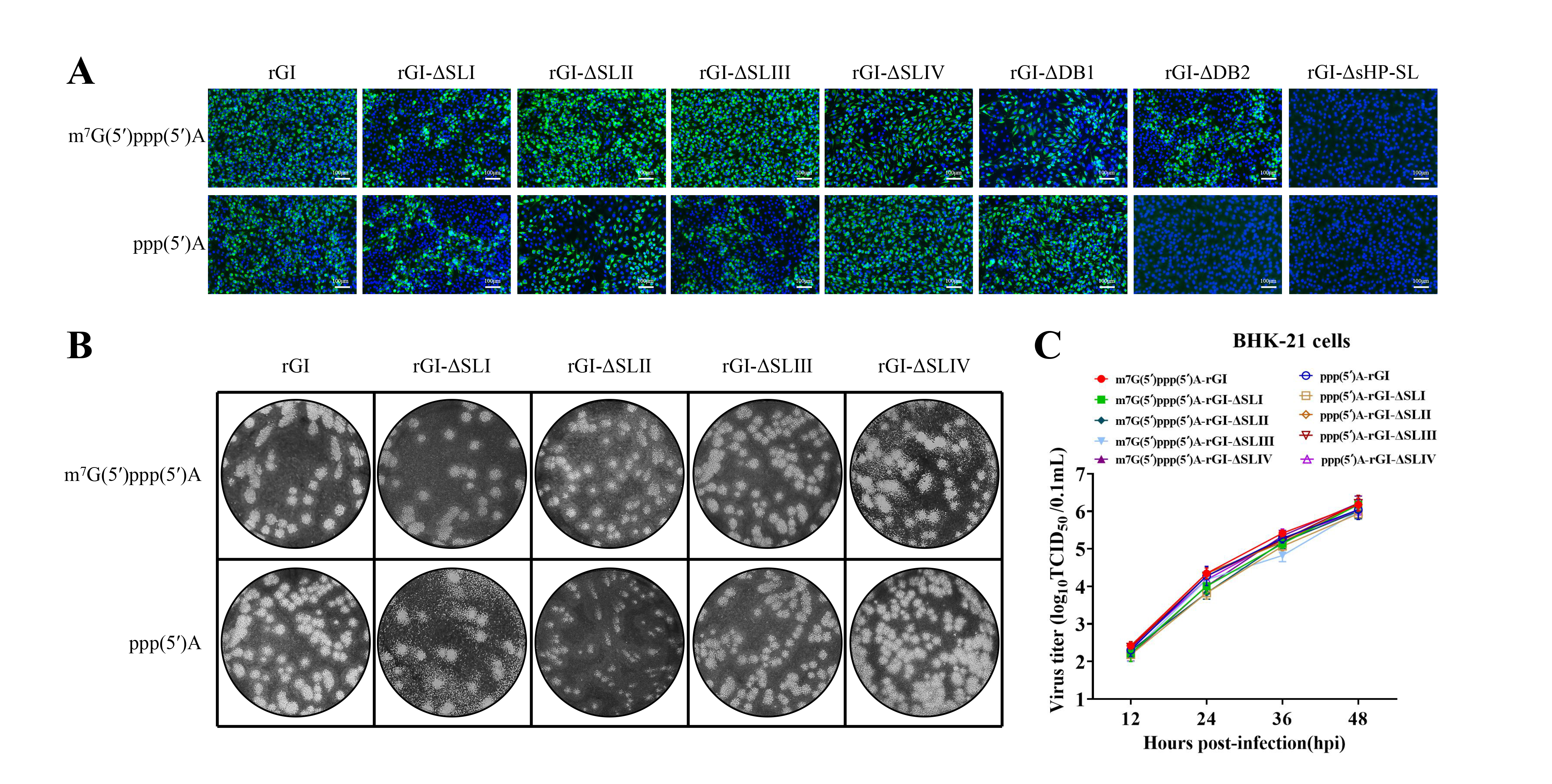
Figure S3.** The immunofluorescence assay and replication characteristics of rescued viruses in BHK-21 cells. A) Immunofluorescence analysis of BHK-21 cells transfected with the JEV genomic RNA with 5′termini m^7^G(5′)ppp(5′)A or ppp(5′)A of WT or deletion mutants. B) Plaque morphologies of the rescued viruses in BHK-21 cells, including rGI, rGI-ΔSLI, rGI-ΔSLII, rGI-ΔSLIII and rGI-ΔSLIV. C) Growth kinetics of the WT and ΔSLI, ΔSLII, ΔSLIII, ΔSLIV mutant viruses in BHK-21 cells. Data are presented as mean ± SD from three independent experiments (C).

**
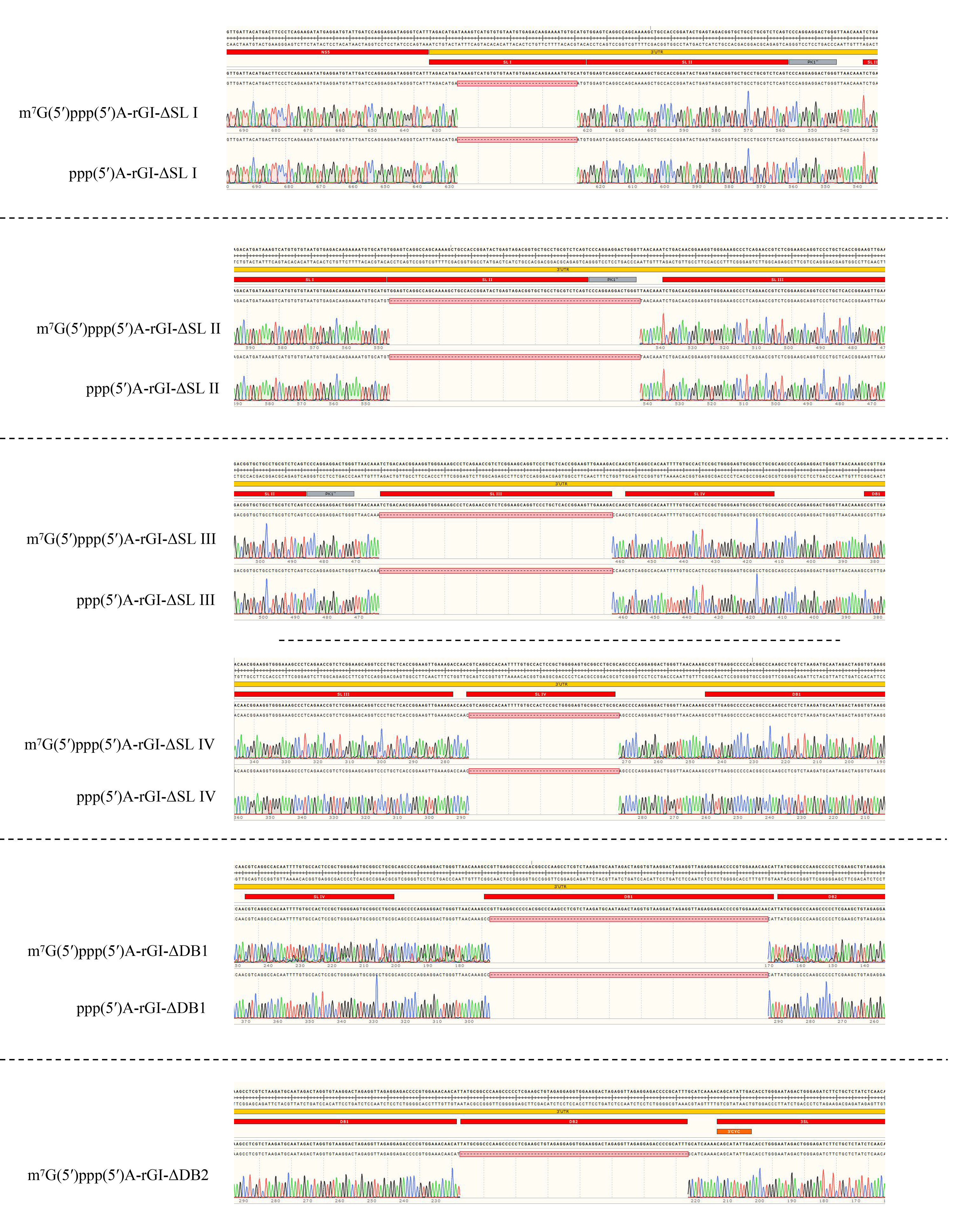
Figure S4.** Sequencing data of deletion mutant viruses.

**
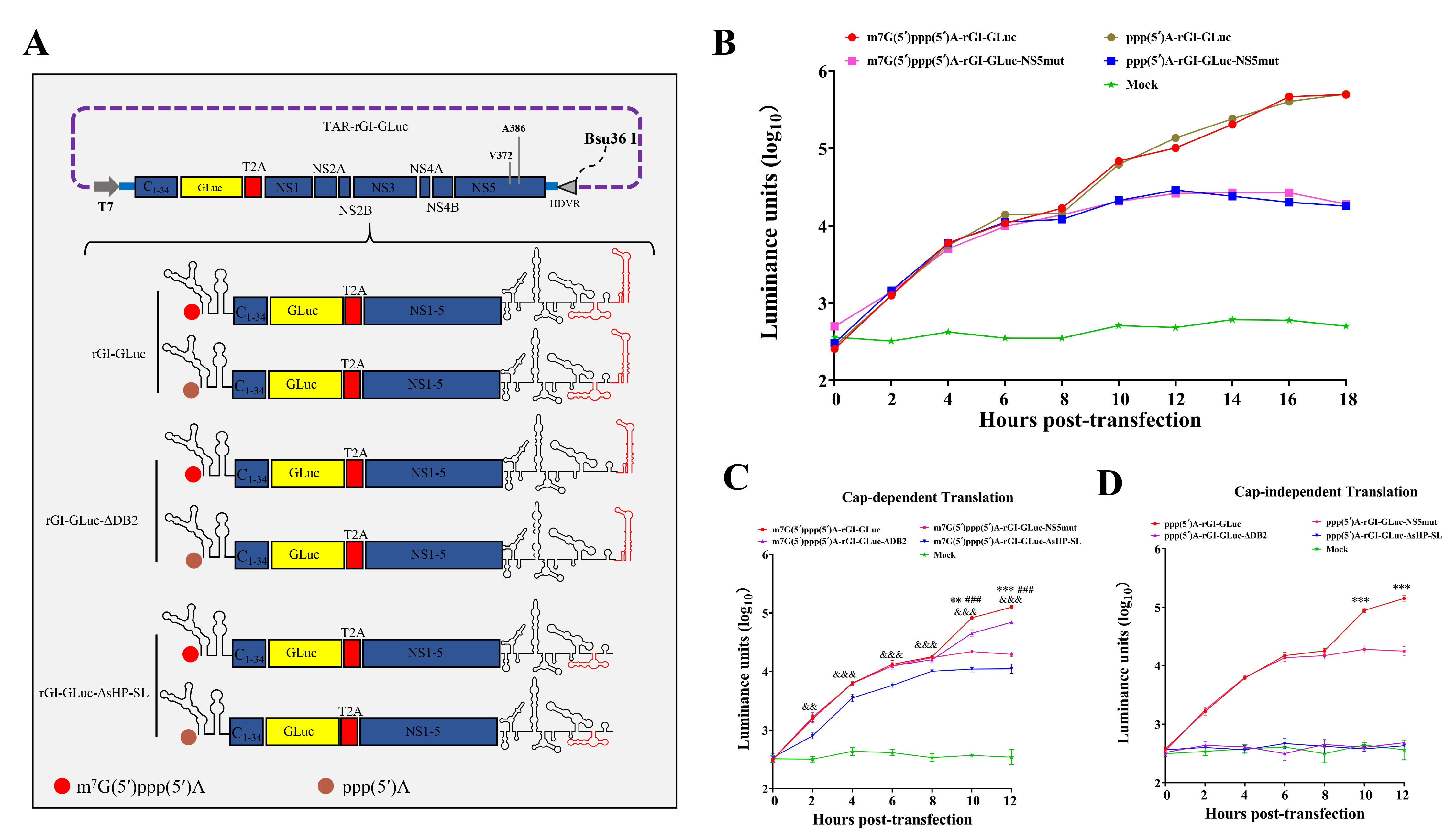
Figure S5.** GLuc-based JEV replicon system for analyzing the translation and replication of viral genomic RNA. A) Schematic diagram of GLuc-based JEV replicon system. B to D) GLuc activities in BHK-21 cells transfected with JEV replicon RNAs or mutants with 5'terminal modified by either m^7^G(5′)ppp(5′)A or ppp(5′). Data are the means ± SD of three independent experiments (C and D). The significant differences between m^7^Gppp(5′)A-rGI-GLuc and m^7^G(5′)ppp(5′)A-rGI-GLuc-ΔsHP-SL are labeled (^&&&^, p<0.001); The significant difference between m^7^Gppp(5′)A-rGI-GLuc and m^7^G(5′)ppp(5′)A-rGI-GLuc-NS5mut is marked (**^###^,** p<0.001); The significant difference between m^7^Gppp(5′)A-rGI-GLuc and m^7^G(5′)ppp(5′)A-rGI-GLuc-ΔDB2, or between ppp(5′)A-rGI-GLuc and ppp(5′)A-rGI-GLuc-NS5mut is marked (***, p<0.001).

**
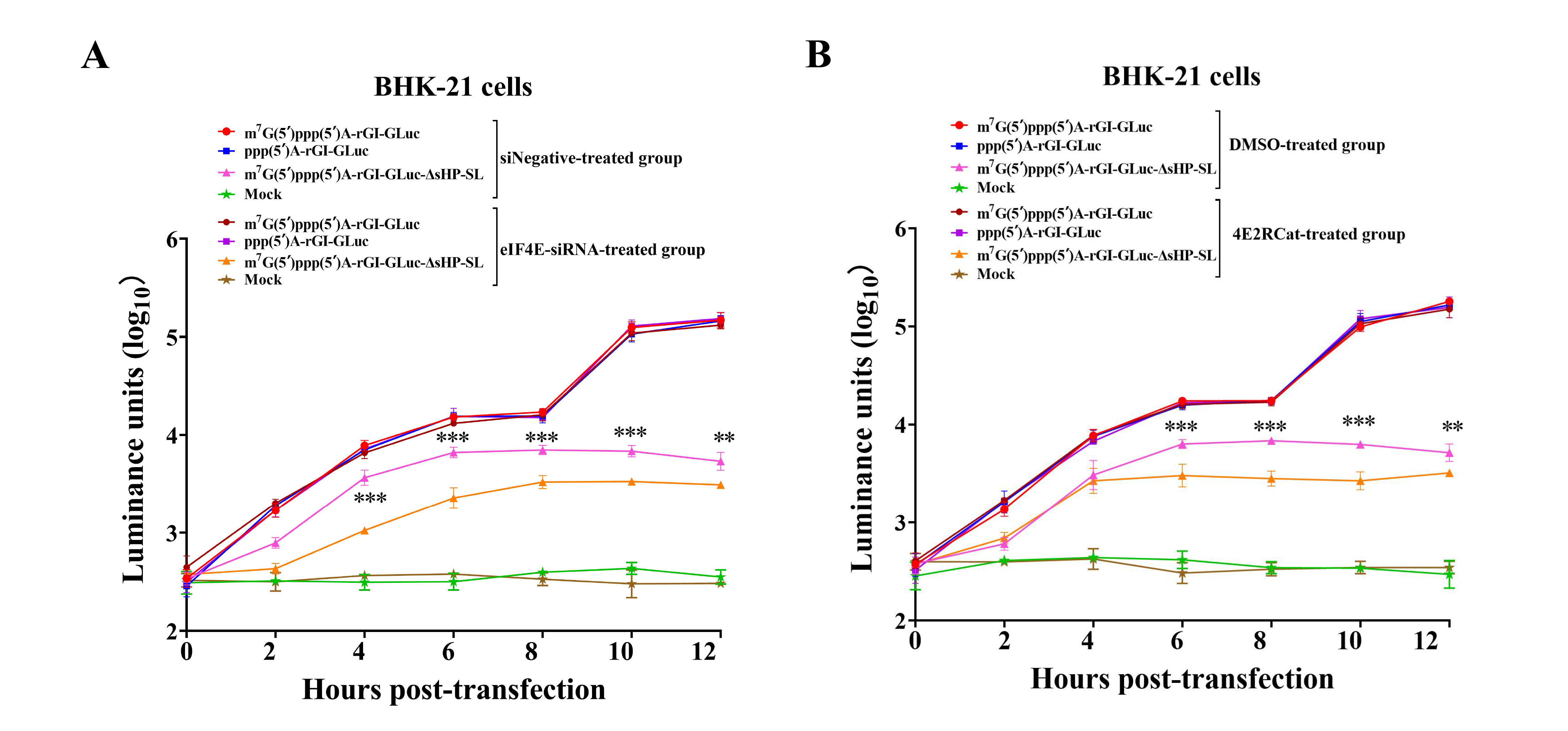
Figure S6.** GLuc-based JEV replicon system for analyzing the translation of viral genomic RNA under the suppression of cap-dependent translation. The BHK-21 cells treated with eIF4E specific siRNA (A) or inhibitor 4E2RCat (B) were respectively transfected with replicon RNAs: [m^7^G(5′)ppp(5′)A]-rGI-GLuc, [ppp(5′)A]-rGI-GLuc, [m^7^G(5′)ppp(5′)A]-rGI-GLuc-ΔsHP-SL to analyze the expression dynamics of GLuc. At different time points after transfection, the supernatants of BHK-21 cells were harvested and mixed with reaction substrate Coelenterazine h (20 µM, pH 7.2) to measure luciferase activity. Data are presented as the mean±SD of three independent experiments. The significant differences of m^7^Gppp(5′)A-rGI-Gluc-ΔsHP-SL between the treated (eIF4E-siRNA or 4E2RCat) and untreated (siNegative or DMSO) groups were labeled (***, p＜0.001; ** p＜0.01).


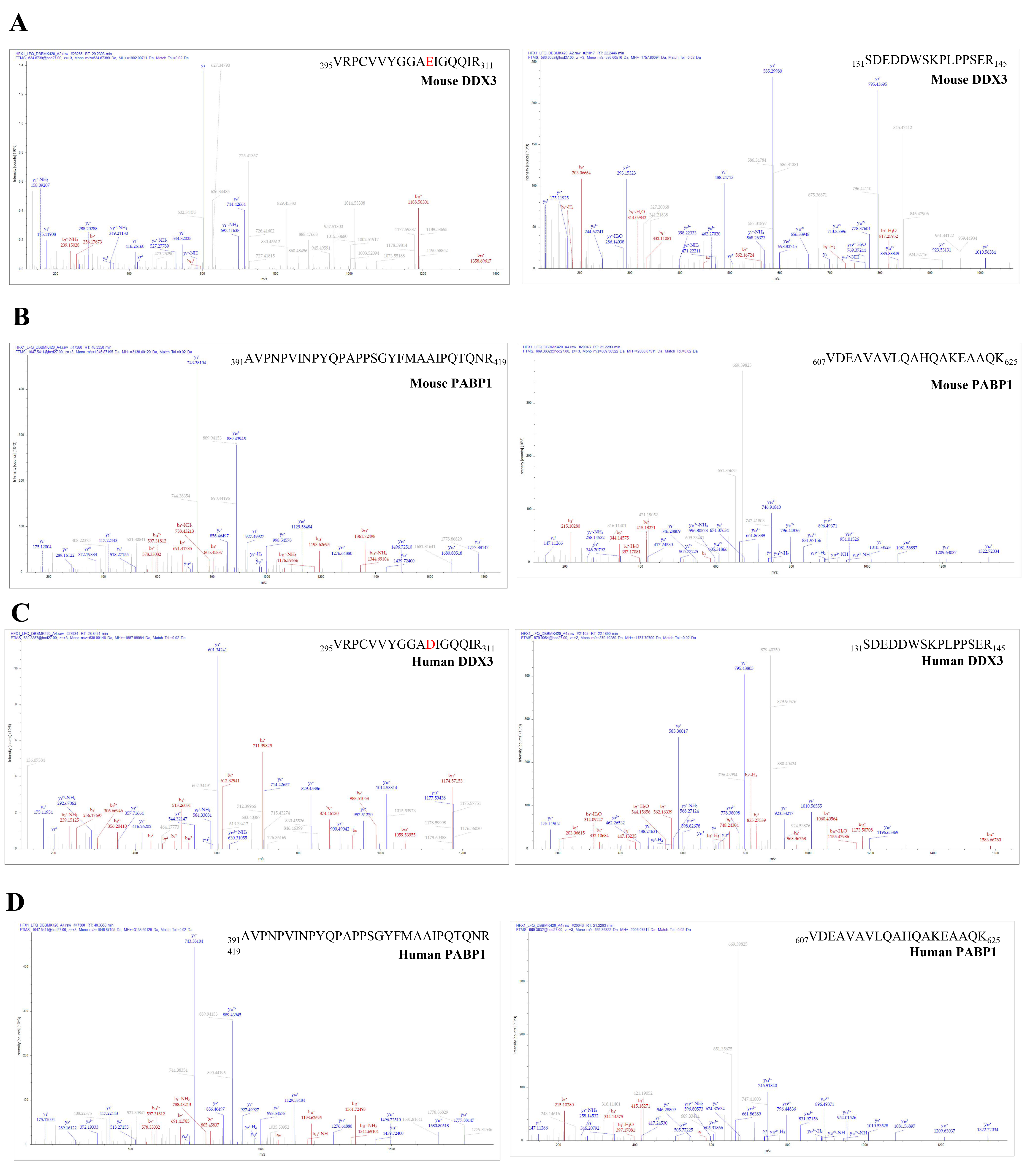
**Figure S7.** Mass spectrometry identification of DDX3 and PABP1 as potential binding partners of JEV 3′UTR. Red indicates matched B ions, blue indicates matched Y ions,grey indicates precursor ions.

**
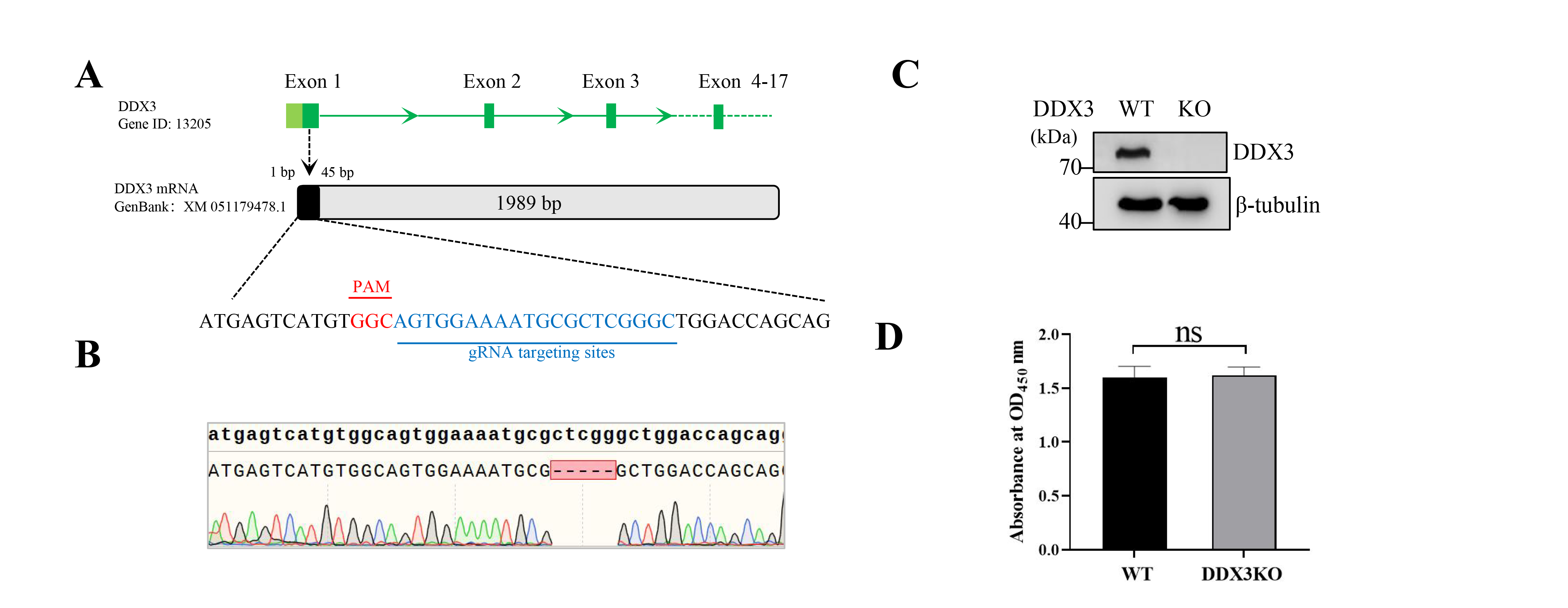
**

**Figure S8.** Generation of DDX3 knock-out BHK-21 cells. A) Schematic illustrating Cas9 inactivation of the hamster DDX3 locus. The 20-bp guide RNA target sequence is shown in blue, and the protospacer-adjacent motif (PAM) is shown in red. B) Identification of the deletion within the DDX3 gene of DDX3-KO BHK-21 cells by DNA sequencing. C) Analysis of DDX3 expression in WT and DDX3-KO BHK-21 cells by immunoblotting. D) Cell proliferation of WT and DDX3-KO BHK-21 cells. ns, no statistical differences. Data are presented as mean ± SD from three independent experiments and tested by Student’s *t*-test (D).

**
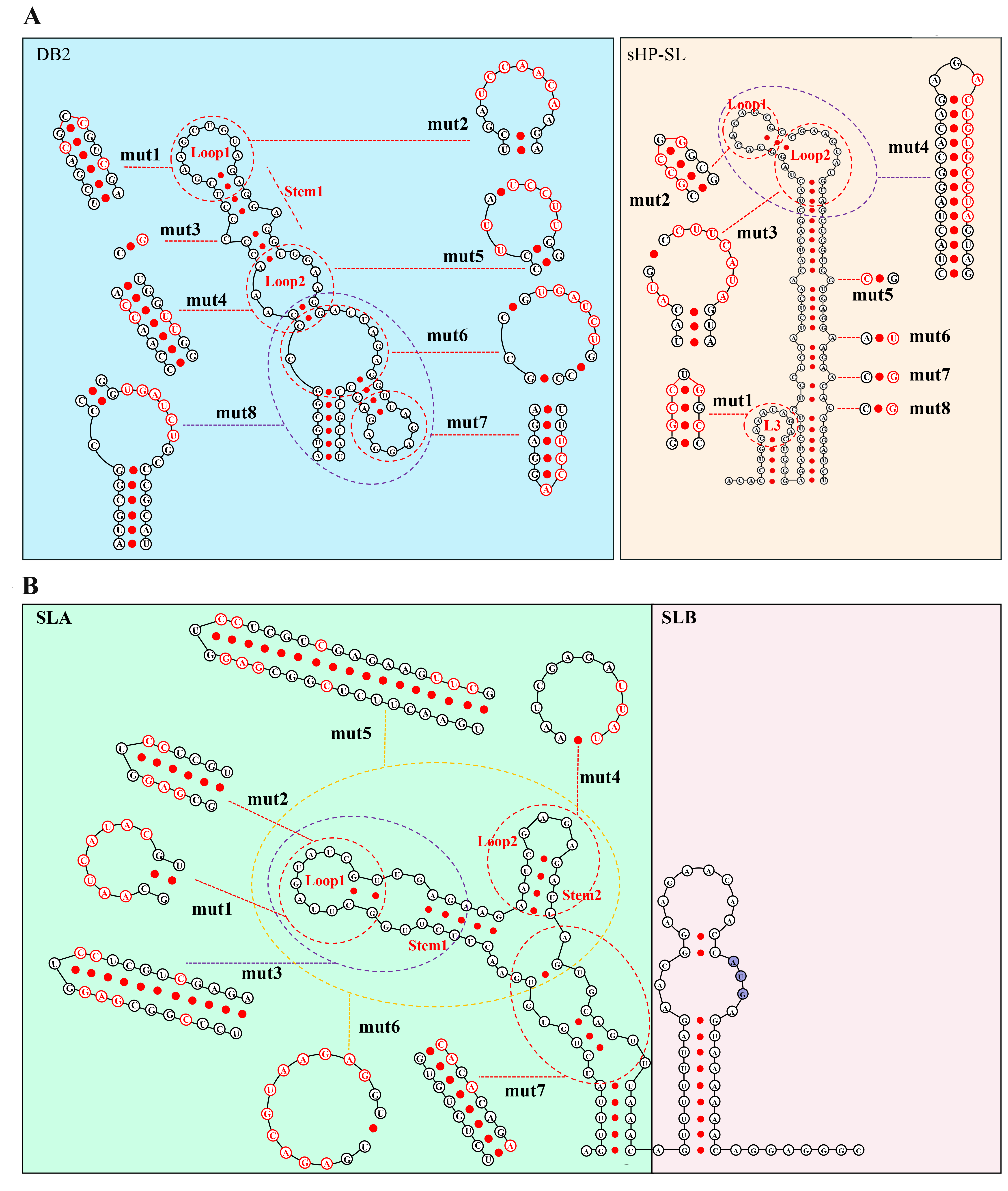
**

**Figure S9.** Identification of the secondary structures within UTRs bound by DDX3 and PABP1. A) Predicted secondary structures of DB2 and sHP-SL in 3′UTR and structures resulting from introduced mutations. Introduced substitutions are shown in red. B) Predicted secondary structures of SLA in 5′UTR and structures resulting from introduced mutations. Introduced substitutions are shown in red.


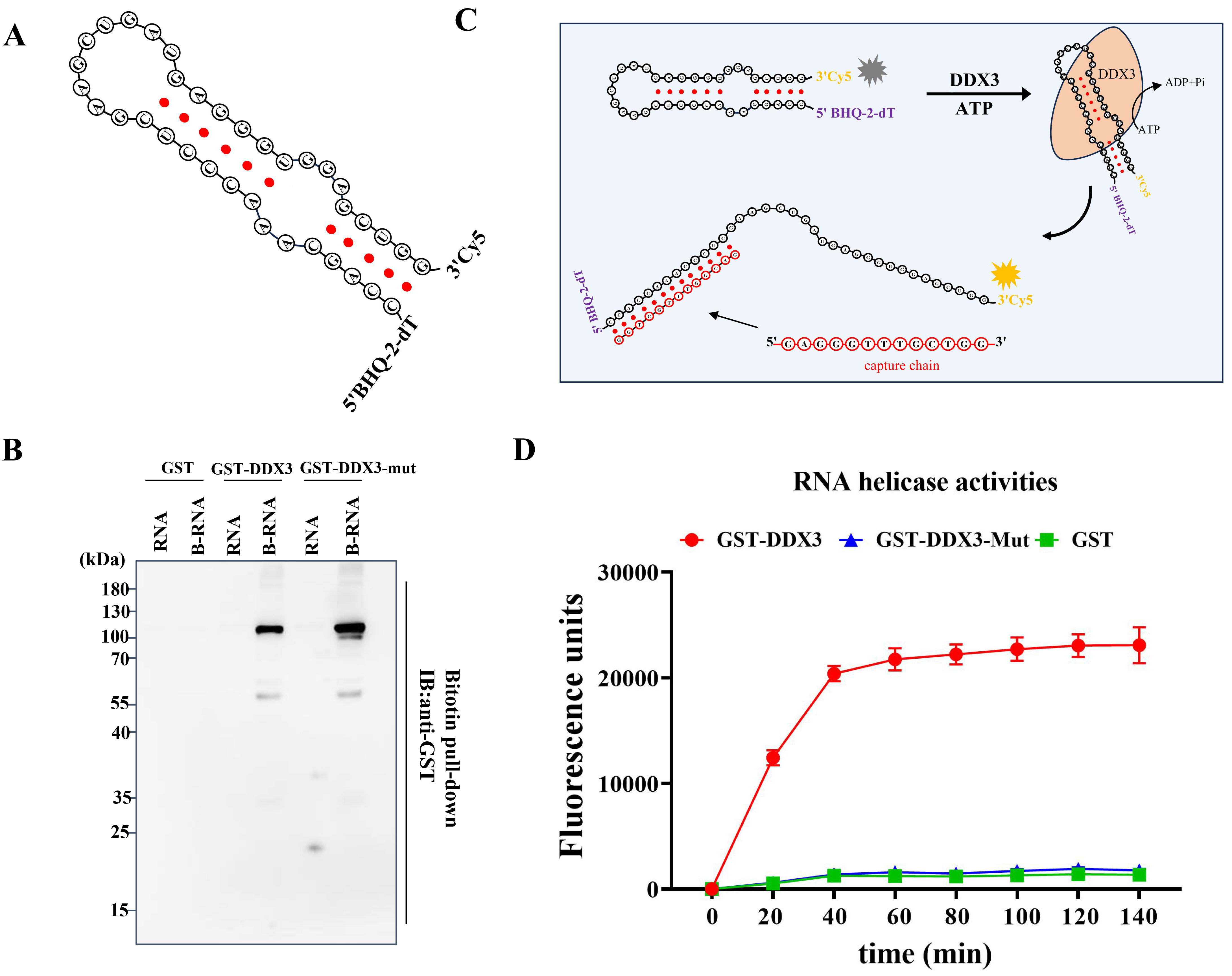


**Figure S10.** The RNA helicase activitiy of DDX3. A) The secondary structure of dsRNA modified with 5′BHQ-2 and 3′Cy5. B) Determination of the binding ability of GST-DDX3 and GST-DDX3 Mut to dsRNA by RNA pull-down assay. C) Schematic diagram of analysis of DDX3 RNA helicase activity based on fluorescence resonance energy transfer.D) Analysis of the helicase activity of GST-DDX3, GST-DDX3-Mut and GS based on FRET. Four replicates from each experimental group were assessed at different time points.

|  | **NCBI description** | **Accession no.** | **Percent sequence coverage** | **Mass (kDa)** |
| --- | --- | --- | --- | --- |
| Human | ATP-dependent RNA helicase DDX5 | NP_001307526.1 | 21 | 69.0 |
|  | Protein arginine N-methyltransferase 5 (PRMT5) | NP_006100.2 | 2 | 72.6 |
|  | ATP-dependent RNA helicase DDX3X (DDX3) | NP_001347.3 | 44 | 73.1 |
|  | ATP-dependent RNA helicase DDX41 | NP_057306.2 | 5 | 69.8 |
|  | Polyadenylate-binding protein 1 (PABP1) | NP_002559.2 | 30 | 70.6 |
|  | Heterogeneous nuclear ribonucleoprotein Q (hnRNP Q) | XP_005248694.1 | 8 | 69.6 |
| Mouse | ATP-dependent RNA helicase DDX5 | XP_051035247.1 | 20 | 69.2 |
|  | Protein arginine N-methyltransferase 5 (PRMT5) | XP_051051682.1 | 11 | 72.7 |
|  | ATP-dependent RNA helicase DDX3X (DDX3) | XP_051035435.1 | 46 | 73.1 |
|  | ATP-dependent RNA helicase DDX41 | XP_051050157.1 | 4 | 71.1 |
|  | Polyadenylate-binding protein 1 (PABP1) | XP_051058699.1 | 28 | 70.6 |
|  | Heterogeneous nuclear ribonucleoprotein Q (hnRNP Q) | XP_051063399.1 | 6 | 69.6 |

Table S1. LC-MS/MS analysis of the cellular proteins associated with elements DB2 and sHP-SL of 3′UTR.

|  | DB2 domain | Viability | | sHP-SL domain | Viability | |
| --- | --- | --- | --- | --- | --- | --- |
|  |  | Cap-dependent | Cap-independent |  | Cap-dependent | Cap-independent |
| Mutation | rGI-3′ UTR-DB2mut1 | + | + | rGI-3′ UTR-SLmut1 | + | + |
|  | rGI-3′ UTR-DB2mut2 | + | + | rGI-3′ UTR-SLmut2 | + | + |
|  | rGI-3′ UTR-DB2mut3 | + | + | rGI-3′ UTR-SLmut3 | + | + |
|  | rGI-3′ UTR-DB2mut4 | + | + | rGI-3′ UTR-SLmut4 | - | - |
|  | rGI-3′ UTR-DB2mut5 | + | + | rGI-3′ UTR-SLmut5 | + | + |
|  | rGI-3′ UTR-DB2mut6 | + | + | rGI-3′ UTR-SLmut6 | + | + |
|  | rGI-3′ UTR-DB2mut7 | + | + | rGI-3′ UTR-SLmut7 | + | + |
|  | rGI-3′ UTR-DB2mut8 | + | + | rGI-3′ UTR-SLmut8 | + | + |
| Deletion | rGI-3′ UTR-ΔDB2 | + | - | rGI-3′ UTR-ΔsHP-SL | - | - |
|  | rGI-3′ UTR-DB2 mutΔ1 | + | - | rGI-3′ UTR-ΔsHP | + | + |
|  | rGI-3′ UTR-DB2 mutΔ3 | + | + | rGI-3′ UTR-SLmutΔ5-8 | + | + |
|  | rGI-3′ UTR-DB2mutΔ5 | + | + | rGI-3′ UTR-SLmutΔ2-3 | + | - |
|  | rGI-3′ UTR-DB2mutΔ7 | + | + |  |  |  |
|  | rGI-3′ UTR-DB2mutΔ1-4 | + | - |  |  |  |

Table S2. The mutant viruses were rescued with capped and uncapped JEV genomes.

+, mutant viruses could be rescued; −, viruses could not be rescued.

| Proteins | Targeting sequences (5'-3') |
| --- | --- |
| Mouse eIF4E | siRNA-1: AGAGUCUAAUCAGGAGGUUGC |
|  | siRNA-2:GGUAUUGAGCCUAUGUGGGAA |
|  | siRNA3:GAUAGUGAUUGGUUACCAGUC |
| Pig eIF4E | siRNA-1: AGCUCACAGAGCUACGAACAG |
|  | siRNA-2:CCUUUGCCUCUGUGGAGCAGU |
|  | siRNA3:ACAAGACUGCCAGCGACCAGG |
| Human/mouse DDX3 | GACGUCUAGUGGAUAUGAUGG |
| Human/ mouse PABP1 | CUGGACUGCUCAGGGUGCCAG |
| Human/mouse DDX5 | CUCCAGAGGGCUAGAUGUGGA |
| Human DDX41 | GCUGCAGGUGCUGCAUUGCG |
| Mouse DDX41 | GGCUGACCGCAUGAUCGACAU |
| Human/mouse hnRNP Q | GCGAGAUGGUGCUGUCAAGG |

Table S3. The siRNAs used for gene silencing in this study.

| Plasmids  Table S4. The primers used for PCR and qPCR in this study. | PCR Primers (5'-3') |
| --- | --- |
| pCMV-Flag-DDX3 | F:CGCGAATTCAATGAGTCATGTGGCAGTGGAAAATGCGCTC |
|  | R:TAGAGTCGACTCAGTTACCCCACCAGTCAACCCCCTGGG |
| pCMV-Myc-DDX3 | F:TCGGTCGACCATGAACCCCAGCGCCCCCAGCTACCCCATGG |
|  | R:CCCGCGGCCGCTTAGACAGTTGGAACACCAGTGGCACTGTT |
| pRL-JEV-5′UTR-FLuc/  pRL-JEV-5′UTR-cHP-cCS-FLuc | F:TCTCAAAAATGAACAATAATTCTAGAAGAAGTTTATCTGTGTGAACTTCT |
|  | R1:GTTTTTGGCGTCTTCCATGGTGGCGCGTTTCAGCATATTGATG |
|  | R2:CGCGTTTCAGCATATTGATGCGCGTTTCAGCATATTGATG |
| pRL-HCV-IRES-FLuc/  pRL-HCV-IRES-ΔdomainIII-FLuc | F:AATGAACAATAATTCTAGAGCCCGCCCCCTGATGGGGGCGACACTCCG |
|  | R:TTGGCGTCTTCCATGGTGGCGCTTCCGCCACCTCCCGGTGGGCG |
| pGL3-JEV-5′UTR-FLuc/  pGL3-JEV-5′UTR-cHP-cCS-FLuc | F:ATCTGCGATCTAAGTAAGCTTAGAAGTTTATCTGTGTGAACTT |
|  | R1:GTTTTTGGCGTCTTCCATGGTGGCGGTTGTTCTTCCGTTCTAAA |
|  | R2:TTCTTTATGTTTTTGGCGTCTTCCATGGTCAGCATATTGATGGCCCGGTT |
| pGL3-JEV-5′UTR-FLuc-3' UTR/  pGL3-JEV-5′UTR-cHP-cCS-FLuc-3' UTR | F:AGATCGCCGTGTAATTCTAGAACATGATAAAGTCATGTGTGTAATGTG |
|  | R:CCGGCCGCCCCGACTCTAGAAGATCCTGTGTTCTTCCTCACCACCA |
| pGL3-HCV-IRES-FLuc/  pGL3-HCV-IRES-Δdomain III-FLuc | F:ATCTGCGATCTAAGTAAGCTTGCCCGCCCCCTGATGGGGGCGAC |
|  | R:GTTTTTGGCGTCTTCCATGGTGGCGCTTCCGCCACCTCCCGGTGGGCG |
| ΔSLI | F:GACATGAATGTGGAGTCAGGCCAGCAAAAGCTGCCACCG |
|  | R:GGCCTGACTCCACATTCATGTCTAAATGACCCTATCCTCC |
| ΔSLII | F:GAAAATGTGCATGTTAACAAATCTGACAACGGAAGGTG |
|  | R:AGATTTGTTAACATGCACATTTTCTTGTCTCACATTACAC |
| ΔSLIII | F:CTGGGTTAACAAACCAACGTCAGGCCACAATTTTGTGCC |
|  | R:GCCTGACGTTGGTTTGTTAACCCAGTCCTCCTGGGACTGAGAC |
| ΔSLIV | F:TGAAAGACCAACAGCCCCAGGAGGACTGGGTTAACAAAGCC |
|  | R:TCCTGGGGCTGTTGGTCTTTCAACTTCCGGTGAGCAGGGA |
| ΔDB1 | F:GGGTTAACAAAGCCCATTATGCGGCCCAAGCCCCCTCGAA |
|  | R:CGCATAATGGGCTTTGTTAACCCAGTCCTCCTGGGGCT |
| ΔDB2 | F:TGGAAACAACATGCATCAAAACAGCATATTGACACCTGGG |
|  | R:GTTTTGATGCATGTTGTTTCCACGGGGTCTCCTCTAACCT |
| ΔsHP-SL | F:ATTTGCATCAAAATGGCCGGCATGGTCCCAGCCTCCTCGC |
|  | R:ACCATGCCGGCCATTTTGATGCAAATGCGGGGTCTCCTC |
| DB2mut1 | F:CCCCTCGACGCCGTCGAGGAGGTGGAAGGACTAG |
|  | R:TCCTCGACGGCGTCGAGGGGGCTTGGGCCGCATA |
| DB2mut2 | F:GCCCCCTCGATCCAACAGAGGAGGTGGAAGGACTAGAGGTTAGAGGA |
|  | R:CTCTGTTGGATCGAGGGGGCTTGGGCCGCATAATG |
| DB2mut3 | F:GATCCAACAGAGGGGGTGGAAGGACTAGAGG |
|  | R:CCTCTAGTCCTTCCACCCCCTCTGTTGGATC |
| DB2mut4 | F:AAGCTGTAGAGGAGGTGGTTGGACTAGAGGTTAGAGGAGACCCCGCATTTGCATCAAA |
|  | R:AACCACCTCCTCTACAGCTTCGAGGGGGTGGTTGGGCCGCATAATGTTGTTTCCACGGG |
| DB2mut5 | F:CCCCCTCGAAGCTGTAGAGGAGGTCCTTGGACTAGAGGTTAGAGGAGACCCCGCATTTG |
|  | R:CCTCTACAGCTTCGAGGGGGTAAGGGCCGCATAATGTTGTTTCCACGGGG |
| DB2mut6 | F:GGAAGGTGATCTGGTTAGAGGAGACCCCGCATTTGCATCA |
|  | R:CTCTAACCAGATCACCTTCCACCTCCTCTACAGCT |
| DB2mut7 | F:GAGGTTTCCAGGAGACCCCGCATTTGCATCAAAACA |
|  | R:CGGGGTCTCCTGGAAACCTCTAGTCCTTCCACCTCCTCT |
| DB2mut8 | F:GGAGGTGGAAGGTGATCTGGCCCGCATTTGCATCAAAA |
|  | R:TTTTGATGCAAATGCGGGCCAGATCACCTTCCACCTCC |
| DB2 mutΔ1 | F:CCAAGCCCCCGGAGGTGGAAGGACTAGAGGTTAGAGGAGACCCCGCATTTGCATCAAAA |
|  | R:TTCCACCTCCGGGGGCTTGGGCCGCATAATGT |
| DB2 mutΔ3 | F:CCCCTCGATCCAACAGAGGGGTGGAAGGACTAGAGGTTAGAGGA |
|  | R:TCCTCTAACCTCTAGTCCTTCCACCCCTCTGTTGGATCGAGGGG |
| DB2 mutΔ1-4 | F:TTATGCGGCCGACTAGAGGTTAGAGGAGACCCCGCATTTGCATCAAAACAGCATATTG |
|  | R:ACCTCTAGTCGGCCGCATAATGTTGTTTCCAC |
| DB2mutΔ5 | F:ATTATGCGGCCGACTAGAGGTTAGAGGAGACCCCGCATTTGCAT |
|  | R:ACCTCTAGTCGGCCGCATAATGTTGTTTCCAC |
| DB2 mutΔ7 | F:GGACTAGAGGCCCCGCATTTGCATCAAAACAGCATATTGACAC |
|  | R:CAAATGCGGGGCCTCTAGTCCTTCCACCTCCTCTACAGCT |
| sHP-SLmut1 | F:TGACACCTGGGCCTGGCCTGGGAGATCTTCTGCTCT |
|  | R:CAGGCCAGGCCCAGGTGTCAATATGCTGTTTTGA |
| sHP-SLmut2 | F:ATCAGCTACTAGGCGCCGGGCGCCGAAGTATGTAGCTGGT |
|  | R:CCCGGCGCCTAGTAGCTGATGTTGAGATAG |
| sHP-SLmut3 | F:GCTACATGGCACAGAGCGCCCTTCATAGTAGCTGGTGGTGAGGAAGAACACA |
|  | R:GGCGCTCTGTGCCATGTAGCTGATGTTGAGATAGAGCAGA |
| sHP-SLmut4 | F:ACAGAGACTGTGCCTATGTAGCTGGTGGTGAGGAAGAACA |
|  | R:TACATAGGCACAGTCTCTGTGCCTAGTAGCTGATGTT |
| sHP-SLmut5 | F:TCTGCTCTATCTCACCATCAGCTACTAGGCACAG |
|  | R:CTGTGCCTAGTAGCTGATGGTGAGATAGAGCAGA |
| sHP-SLmut6 | F:CTGGTGGTGAGGTAGAACACAGGATC |
|  | R:GATCCTGTGTTCTACCTCACCACCAG |
| sHP-SLmut7 | F:TGTAGCTGGTGGTGAGGTAGAACACAGGATCT |
|  | R:AGATCCTGTGTTCTACCTCACCACCAGCTACA |
| sHP-SLmut8 | F:TGTAGCTGGTGGTGAGGAAGAACAGAGGATCT |
|  | R:AGATCCTCTGTTCTTCCTCACCACCAGCTACA |
| ΔsHP | F:CATATTGACAAGATCTTCTGCTCTATCTCAACA |
|  | R:CAGAAGATCTTGTCAATATGCTGTTTTGATGCAAAT |
| sHP mutΔ5-8 | F:AGCGCCGAAGTATGTAGCTGGTGTGAGGAGACAAGGATCT |
|  | R:AGATCCTTGTCTCCTCACACCAGCTACATACTTCGGCGCT |
| sHP mutΔ2-3 | F:CATCAGCTGTAGCTGGTGGTGAGGAAGAACACAG |
|  | R:CCACCAGCTACAGCTGATGTTGAGATAGAGCAGAAGA |
| SLA-mut1 | F:TTCTTGGCAATCATACGTTGAGAAGAATCGAGAGATTAG |
|  | R:ACGTATGATTGCCAAGAAGTTCACACAGATAAAC |
| SLA-mut2 | F:TTGGCGAGGTCCTCGTTGAGAAGAATCGAGAGATTA |
|  | R:CAACGAGGACCTCGCCAAGAAGTTCACACAGATAAAC |
| SLA-mut3 | F:CGGCGAGGTCCTCGTCGAGAAGAATCGAGAGATTAGTGCAGTTTAAACAGTT |
|  | R:TCGACGAGGACCTCGCCGAGAAGTTCACACAGATAAACTTCT |
| SLA-mut4 | F:AAGAATCGATTATTAGTGCAGTTTAAACAGTT |
|  | R:CACTAATAATCGATTCTTCTCAACGATACTAAGCCAAG |
| SLA-mut5 | F:TCTCGGCGATGTCATCGTCGAGAAGTTCGTGCAGTTTAAACAGTTTTTTAGAA |
|  | R:ACGATGACATCGCCGAGAAGTTCACACAGATAAACTTCT |
| SLA-mut6 | F:TGAGACGTAAGAGGTTGCAGTTTAAACAGTTTTTTAG |
|  | R:ACCTCTTACGTCTCACACAGATAAACTTCT |
| SLA-mut7 | F:GAGATTACACACAGATTAAACAGTTTTTTAGAACGGAA |
|  | R:TAATCTGTGTGTAATCTCTCGATTCTTCTCAACGATACTAAG |
| SLA mutΔ1 | F:AACTTCTTGTGAGAAGAATCGAGAGATTAGTGCAG |
|  | R:GATTCTTCTCACAAGAAGTTCACACAGATAAACTTCT |
| SLAmutΔ3 | F:AGAAGTTTATCTGTGTGAACTAGAATCGAGAGATTAGTGCAGTTTAA |
|  | R:TTAAACTGCACTAATCTCTCGATTCTAGTTCACACAGATAAACTTCT |
| SLAmutΔ4 | F:TGAGAAGAATCGATAGTGCAGTTTAAACAGTTTTTTAGAA |
|  | R:AACTGCACTATCGATTCTTCTCAACGATACTAAGCCAAGAAG |
| SLAmutΔ6 | F:AGAAGTTTATCTGTGTGCAGTTTAAACAGTTTTTTAGAACGGA |
|  | R:TCCGTTCTAAAAAACTGTTTAAACTGCACACAGATAAACTTCT |
| SLAmutΔ7 | F:AAGAATCGAGAGATTATTAAACAGTTTTTTAGAACGGAAGAAC |
|  | R:ACTGTTTAATAATCTCTCGATTCTTCTCAACGATACTAAGC |
| PCR-based reverse genetics system for DTMUV | PCR Primers (5'-3') |
| DTMUV-T7-1F | GCTAATACGACTCACTATAGGAGAAGTTCATCTGTGTGAACTTATTCCAAACAGCTTTT |
| DTMUV-2406R | TCATAGAAATGGATCTGTCCCTTGCATTCAGCCCCATC |
| DTMUV-2366F | GTGGATGGGGCTGAATGCAAGGGACAGATCCATTTCTA |
| DTMUV-5092R | CCTTGAGTGATCATACTGACAAAATCGCCTGAACCCACCAGGA |
| DTMUV-5025F | TGGTTGGCCTCTATGGTAATGGAATCCTGGTGGGTTCAGG |
| DTMUV-8318R | CCAGTACATCTCATGTGTTGAATTACGCGAGAGTGGCACCCTA |
| DTMUV-8258F | TGGGGAGGTGGTTTGGTTAGGGTGCCACTCTCGCGTAAT |
| DTMUV-10991R | AGACTCTGTGTTCTACCACCACCAGCCACACTTTCGGCGATCTG |
| DTMUV-ΔDB2-F | GGGACTAGAGACTAGAGGTTAGAGGAGACCCCTTGCGAGT |
| DTMUV-ΔDB2-R | AACCTCTAGTCTCTAGTCCCTCCATGATGGTCACACCAGC |
| DTMUV-sHP-SL | TCACTCGCAAGGGGTCTCCTCTAACCTCTAGTCTCT |
| Gene name | qPCR primers (5'-3') |
| JEV | F:GGGTCAGATCCGTCACTAGAC |
|  | R:ACGACGAACGTGGAGTTGGC |
| Mouse β-actin | F:CGGCATTGTCACCAACTGGG |
|  | R:GCCTGAATGGCTACGTACAT |
